# Health risk behaviours among adolescents in the English-speaking Caribbean: a review

**DOI:** 10.1186/1753-2000-3-10

**Published:** 2009-03-17

**Authors:** Rohan G Maharaj, Paula Nunes, Shamin Renwick

**Affiliations:** 1 Unit of Public Health and Primary Care, Faculty of Medical Sciences, The University of the West Indies, St. Augustine, Trinidad and Tobago; 2 Medical Sciences Library, Faculty of Medical Sciences, The University of the West Indies, St. Augustine, Trinidad and Tobago

## Abstract

**Background:**

The aim of this paper was to review and summarize research on prevalence of health risk behaviours, their outcomes as well as risk and protective factors among adolescents in the English-speaking Caribbean.

**Methods:**

Searching of online databases and the World Wide Web as well as hand searching of the *West Indian Medical Journal *were conducted. Papers on research done on adolescents aged 10 – 19 years old and published during the period 1980 – 2005 were included.

**Results:**

Ninety-five relevant papers were located. Five papers were published in the 1980s, 47 in the 1990s, and from 2000–2005, 43 papers. Health risk behaviours and outcomes were divided into seven themes. Prevalence data obtained for these, included lifetime prevalence of **substance use**: cigarettes-24% and marijuana-17%; **high risk sexual behaviour**: initiation of sexual activity ≤ 10 years old-19% and those having more than six partners-19%; **teenage pregnancy**: teens account for 15–20% of all pregnancies and one-fifth of these teens were in their second pregnancy; **Sexually-Transmitted Infections (STIs)**: population prevalence of gonorrhoea and/or chlamydia in 18–21 year-olds was 26%; **mental health**: severe depression in the adolescent age group was 9%, and attempted suicide-12%; **violence and juvenile delinquency**: carrying a weapon to school in the last 30 days-10% and almost always wanting to kill or injure someone-5%; **eating disorders and obesity**: overweight-11%, and obesity-7%. Many of the risk behaviours in adolescents were shown to be related to the adolescent's family of origin, home environment and parent-child relationships. Also, the protective effects of family and school connectedness as well as increased religiosity noted in studies from the United States were also applicable in the Caribbean.

**Conclusion:**

There is a substantial body of literature on Caribbean adolescents documenting prevalence and correlates of health risk behaviours. Future research should emphasize the designing and testing of interventions to alleviate this burden.

## Background

The seventeen English-speaking Caribbean territories referred to in this article, have similar political, social, educational and cultural systems as a result of having a British colonial background. These are Anguilla, Antigua and Barbuda, The Bahamas, Barbados, Belize, Cayman Islands, Dominica, Grenada, Guyana, Jamaica, Montserrat, Saint Kitts and Nevis, Saint Lucia, Saint Vincent and the Grenadines, Trinidad and Tobago, Turks and Caicos, and the British Virgin Islands.

Though the countries may be separated by seas, most are small island economies (except Belize and Guyana) which share a historical past that left an ethnic mix with descendants of a European upper class, African slaves, and migrant labourers from countries like India and China.

Currently about half of the world's population is under the age of 25. Similarly, in the English-speaking Caribbean countries, adolescents represent about 20% of the population, or approximately 1.2 million persons (1 224 720 out of 6 161 910) according to 2007 population data[1].

Although the overall mortality rate in adolescents is low (70/100 000 for Latin America and the Caribbean [2]), as of the year 2000, the major causes of mortality among 15–24 year-olds are homicide, accidents and suicide, followed by Acquired Immune Deficiency Syndrome (AIDS) [3]. Additionally, adolescent obesity is on the rise in the Americas with 8–22% of adolescents being obese [4]. These trends are a source of great concern as it is during this period that lifestyle choices are made which determine the eventual burden on health care systems. Around half of all preventable premature adult deaths are attributable to acquired risk factors dating back to adolescence, such as, smoking, poor eating habits, and a lack of physical exercise [4]. It is through the understanding of the health risk and protective factors as well as the postulates of researchers in the region that interventions may be designed and implemented which could impact positively on the health, quality of life and productivity of our Caribbean societies.

In a preliminary review on the health behaviour of the adolescent in the region, no papers were identified prior to 1980; therefore this review covers the literature over the consequent twenty-five years, 1980 – 2005. It is hoped that in reviewing this extensive period Caribbean researchers would become aware of the wealth of data that is available on the prevalence of health risk behaviour, their outcomes and protective factors affecting Caribbean adolescents and, as such, can develop community effectiveness and efficacy trials to address this important segment of the population [5].

## Methods

A review of the health literature published on adolescents in the English-speaking Caribbean was conducted. A combination of online searching of bibliographic databases and the World Wide Web as well as hand searching of individual issues of the *West Indian Medical Journal*, its supplements (which contain the abstracts of regional research meetings) and its annual indexes (found in the December issues) from 1992 to 2005 was accomplished.

The following inclusion criteria were used: the paper (1) must be in the form of an abstract, thesis, local country report or published in a peer-reviewed journal; (2) must deal with or contain information on adolescents between ages 10–19 years old from the English-speaking Caribbean; (3) must contain information on prevalence data on health risk behaviours, outcomes, risk or protective factors; and (4) must be in English and published during the period January 1980 to November 2005.

Several papers identified were available only in abstract form, especially those from Caribbean Commonwealth Medical Research Council (CCMRC) and the Caribbean Health Research Council (CHRC) conferences. Many of these papers have not been published in full-text formats. Country reports were included as sometimes they were the only source of relevant information available.

## Results

A total of 95 relevant papers were identified) [6-100]. Additional file 1 provides a summary of these papers and their contents.

Five papers were published in the 1980s, 47 papers in the 1990s, and 43 papers from 2000–2005. Thirty-four papers were published internationally, 55 regionally (i.e. within the Caribbean) and six locally as country reports. There were 58 full-text publications (51 full-text journal articles, five local reports, one book and one book chapter). And for 28 papers, only abstracts were available. Nine relevant theses were located at the libraries of The University of the West Indies.

The methodologies employed in the studies included surveys (65), retrospective reviews of case records (15), interviews (8), case-controlled studies (6), focus groups (5), secondary review of previously collected data (3), prospective autopsy study (1) and cohort study (1). Ten publications used more than one methodology.

From the research, health risk behaviours and outcomes identified could be grouped into seven main categories: substance use, high risk sexual behaviour, teenage pregnancy, STIs, including Human Immunodeficiency Virus (HIV)/AIDS, mental health, violence and delinquency, and eating behaviours and obesity.

### Substance Use

There were 21 papers, starting from the early 1990s, which dealt primarily with substance use. There were nine full-text peer-reviewed papers; seven published only as abstracts; two country reports; two theses; and one chapter in a book.

#### Prevalence studies

Most papers provided prevalence data with the common indicators: 30-day prevalence and lifetime prevalence. Although papers differed methodologically, alcohol was the most commonly used substance followed by cigarettes and then marijuana. Additional file 1 summarises the general substance use [6-11] and 30-day prevalence and the life-time prevalence of substance use for selected drugs [12-28].

#### Risk factors for substance use

Several Caribbean studies identified the following risk factors for substance use: being male [12-15], having a family member using or supporting the adolescents' use of the substance [6,10,12,14], absence of religious involvement [6,12,16], having lower grades at school [6,12], having larger amounts of spending money [6,12] and being children of professionals [13].

In addition, the *Caribbean Youth Health Survey *reported that abuse, skipping school and experiencing rage [9] were risk factors for smoking and alcohol use. A study done in Trinidad and Tobago, where the ethnic mix of persons of East Indian descent (Indo-Trinidadian) to African descent (Afro-Trinidadian) is about equal (this mix being similar only to Guyana as in the other countries there is a majority of persons of African origin), found that Indo-Trinidadian adolescents were more likely to have used alcohol in the last month while Afro-Trinidadian adolescents were more likely to have used marijuana [6,17].

### High risk sexual behaviour

Twenty-two papers addressing high risk sexual behaviour were identified. There were 13 full-text papers, four abstracts, three local reports and two theses. Four themes were noted in these papers: (a) studies looked at the prevalence of high-risk sexual behaviours (reported age of sexual debut, presence of multiple partners, and lack of contraceptive or condom use); (b) risk factors; (c) protective factors for initiating sexual activity; and (d) teenage pregnancy, HIV/AIDS and STIs.

#### Prevalence of common high-risk sexual behaviours among Caribbean adolescents

Sixty-six percent of adolescents reported that they had not had sexual intercourse [10]. The papers reporting prevalence of high-risk sexual behaviour, including initiation of sexual activity before the age of 10 years, not using a contraceptive method, having multiple sexual partners in the past 12 months, having more than six sexual partners and participating in anal sex are presented in Additional file 1[15,19,22,23,25,29-42].

#### Risk factors for early initiation of sexual activity

Of the adolescents who had early initiation of intercourse, many (38%) indicated that the initial encounter was forced. Indeed a history of physical or sexual abuse was found to be a predictor of having sexual intercourse as an adolescent [10,22]. Additional risk factors were 'less family stability', single-parent family households, low socioeconomic status, and poor knowledge of STIs [43] as well as male gender, recent substance use, recent depression or attempted suicide [22]. Higher levels of sexual activity were reported if there was little adult supervision, adolescents had no specific household chores or homework or sleeping facilities were shared [44]. In females, increased parity and experiencing menarche at an earlier age were also associated [43].

#### Protective factors for sexual activity

Protective factors included a good relationship with parents, involvement in extracurricular activities, and attending church [29]. Family connectedness [40] and attending church [9,29,32,33,45] were also protective in delaying sexual debut. Adolescents who liked school were less likely to report fear or concerns about the consequences of sexual activity as their reasons for delayed coitus. In addition, those who attended religious services as well as had married parents were significantly less likely to also cite the "lack of opportunity to have sex" as an explanation for not being sexually active [32]. In Anguilla, the top three reasons for abstaining from sexual activity included "wanting to wait until older", "no opportunity with someone I like" and "not being emotionally ready" [22].

### Teenage Pregnancy

Several misconceptions about pregnancy were noted among adolescents with approximately one third being unaware that pregnancy was possible at first intercourse. Many males believed that having sex while standing prevents pregnancy, and that condoms were only for boys who have sex with more than one girl [33].

Fourteen papers were located which dealt with risk factors and pregnancy outcomes in adolescents. There were seven full-text publications, five abstracts and two theses. Research in this area focused on four themes: (a) the risk factors contributing to teen pregnancy; (b) the prevalence of teen pregnancy; (c) the risk and complications of teen pregnancy, and (d) repeat pregnancy among teens.

#### Risk factors for teen pregnancy

Four papers addressed the issue of risk factors for teen pregnancy. One conclusion arising out of these papers suggests that teens who got pregnant, themselves had teenage mothers [46-48]. These teens either lived in homes with no male authority or father figure [46,49] or tended to live away from their parents [48]. There was also a higher likelihood that the adolescent had been sexually active before age 16 [46] and had never had discussions with their parents about sexuality [48]. In 1999, it was observed that most teenage pregnancies occurred in unmarried females and, if married, the teenagers were in unstable relationships with high rates of divorce [50].

#### The prevalence of teen pregnancy

Despite the early initiation of sexual activity among teens in the Caribbean there is growing evidence of falling adolescent birth rates. For example, in Antigua and Barbuda, there was a 43% decrease in all adolescent births between the periods 1969–73 and 1994–8 [51] and in Trinidad, a 2% decrease between 1960 and 1987 [52]. Overall, teenage pregnancies represented 15% – 20% of all pregnancies [53,54].

#### Risk and complications associated with teen pregnancy

Teen pregnancies have an increased risk of complications which include: preterm labour [54], operative delivery [54,55], small for gestational age babies, prematurity and perinatal mortality [53,54,56], ante-partum and post-partum haemorrhage, elevated blood pressure, pre-eclampsia, eclampsia, prolonged rupture of membranes and prolonged labour [55]. Where antenatal care for teenage pregnancies is high, the 'obstetrical performance' (as measured by antenatal and intra-partum complications) was similar to matched controls [57,58].

#### Repeat pregnancy among teens

The risk predictors of one or more repeat pregnancies were common-law relationships with either the father of the first baby or another current partner, perceptions of one's socioeconomic status as very poor or poor and being a member of household where the respondent or spouse was the main wage earner. Variables that exerted a protective effect against the occurrence of one or more repeat pregnancies were: the desire to continue one's education after the birth of first child, taking action to continue education, use of contraception after first birth, being a member of a household in which the mother was the major wage earner at the time of the first birth and the absence of a current sexual relationship with their first 'baby father' [59].

### STIs and HIV/AIDS

The area of STIs including HIV/AIDS is one of increasing interest to researchers in the field of adolescent health and much research has been carried out since 2000. Fifteen papers (nine full-text articles, three reports, two abstracts and one thesis) cover this topic.

#### Risk factors for STIs

Multiple partners, low frequencies of condom use in the last sexual encounter or among those with multiple sexual partners, marijuana use and having multiple sexual partners were some of the common risk factors identified for STIs [15,19,37,60]. An increased risk of HIV occurred in individuals who had a history of genital ulcer disease and gonorrhoea [37]. In different populations there are other psychocultural issues which have been identified, such as, infidelity, sex-in-exchange for resources and lack of frank discussions on sexual issues which is thought to contribute to the HIV epidemic in the region [61].

#### Factors protecting against STIs

Increased educational achievement, consistent condom use and delaying the age of sexual debut were all identified as protective factors against STIs; for every year increase in level of education, the odds of reporting STI symptoms decreased by 0.87 [37,62]; and for every year increase in the age of first intercourse, the odds of reporting STI symptoms decreased by 0.92. Males who reported consistent condom use with steady partners were less likely to report symptoms of STIs than were inconsistent users [37].

#### HIV/AIDS

The Caribbean literature identified focussed on adolescent perceptions of HIV/AIDS. Again even though not dealing with behaviours the relevant research has important implications for risk behaviours and is, therefore, included in this review. As was noted above Caribbean adolescents are aware of HIV and AIDS, with as many as 86% having heard about AIDS, and 90% knowing that HIV was sexually transmitted [44]. Young persons 10–20 years old indicated that they were "afraid of getting AIDS" [22]. A report on HIV infection among adolescents in Jamaica found that the mean age of diagnosis was 15.6 years [63]. The cumulated case rate for HIV in Jamaica between 1982 and 2001 for 10–19 year olds was 10/100 000 males and 27/100 000 females. Consensual sex was the most common method of transmission in 56% of cases; in another study among adolescent attendees at an STI clinic, co-infection with HIV was noted in one percent of attendees[19].

One paper, which studied Jamaican street boys between the ages of 11 and 17, identified the following risk factors for HIV: an inability to obtain condoms; negative attitudes toward condom use; early age of sexual initiation; multiple sex partners; as well as drug and alcohol use. In addition, many of these boys held misconceptions about HIV/AIDS. Other issues identified included intolerance toward homosexual behaviour and physical abuse against girls [64].

Much of the work on HIV/AIDS has been conducted by regional organisations. Research such as KAPB (Knowledge, Attitude, Practices and Behaviour) studies of the general population has not been published in complete form internationally. These are represented in Additional file 1[65-67]. Additional information regarding sexually transmitted diseases among adolescents and young people in the Caribbean is also provided in Additional file 1[15,19,37,60,62,68].

### Mental Health

A total of 18 items were found: ten full-text papers, six abstracts, one report and one thesis. Papers dealt primarily with psychopathology [69,70], attempted suicide (parasuicide) and suicide [71-80] as well as depression [81-84].

#### Psychopathology

Fear of injury or death of self or loved one, sexual issues and failure at school were the major concerns of adolescents [69]. Females were also more likely to have experienced an adolescent crisis, while male adolescents were more often diagnosed with schizophrenia. Psychosexual problems, parental conflict and hostility were the main risk factors for these psychopathologies [70]. An increased prevalence of health compromising behaviours were noted in adolescents who experienced physical or sexual abuse and in those who had a friend or relative who had attempted suicide [10]. Reported protective factors for these psychopathologies were avoiding parental separation, divorce or the absence of one parent [70].

#### Attempted suicide

Corresponding with international data, females had higher rates of attempted suicide [71,72,75,76]. The main reason given for attempting suicide was interpersonal conflicts which included intra-familial and marital conflicts as well as lovers' quarrels. Alcohol use with prior or attempted suicide was also noted [71]. There were ethnic differences in Trinidad where Indo-Trinidadians made more suicide attempts than Afro-Trinidadians or mixed race counterparts [72-75]. Among hospital admissions, 25% were found to be depressed and 22% had adjustment disorders [73]. In Guyana, a similar ethnic difference was reported [74]. In South Trinidad most patients came from rural areas and identified family instability, emotional problems, financial difficulties, peer pressure, and unemployment as additional risk factors for attempting suicide [75].

The most common method of attempting suicide was by ingestion of a toxic substance, mainly, herbicide (paraquat) (63%) or insecticide (organophosphates) (20%). Intake of oral medication to commit suicide was about 8% [73,76]. The main strategies used for healing were family support and counselling [75,76].

#### Completed suicide

The only papers concerning completed suicide came from Trinidad and Tobago. Of 270 cases of completed suicide reported at the General Hospital in Port-of-Spain, Trinidad, 10% were from the 11–18 year group compared to the 19–26 year olds who had the highest number of cases (25%). The ethnic base of this sub-population had equal numbers of male patients of African and East Indian descent; however, in females, Indo-Trinidadian patients outnumbered Afro-Trinidadian patients by two to one. Lovers' quarrels, psychiatric illness and family disputes accounted for the majority of cases. Persons of Indo-Caribbean origin predominated in suicides due to lovers' quarrels or family disputes [78,79] and persons of Afro-Caribbean origin were slightly (53% vs. 45%) more represented in persons suffering from psychiatric illnesses. Depression was the most common psychiatric illness diagnosed. The herbicide, paraquat, was the most commonly used substance in both North and South Trinidad [78-80].

#### Depression

Depression was twice as likely to occur in females as males (18% vs. 8%) with the highest rate of depression in the 16 to 17-year group. Attendance at a religious institution and prayer with the family was associated with a lower depression rate. Intact families had the lowest rate (12%), while the reconstituted family had the highest rate (26%). Adolescents were more likely to be depressed if there was abuse of alcohol among family members and if they attended schools which had low status ranking in terms of academic performance [67]. There were no ethnic differences among depression cases. A review of the impact of protective factors showed that attendance at a religious institution lowered only suicidal ideation, while prayer with the family lowered both suicidal ideation and suicide attempts. Individuals with alcohol abuse in the family had higher suicidal ideation and attempts [81]. Depression rates among adolescents ranged from 9–28%, however, these rates include the spectrum of mild to severe depression [81-84]. Psychological issues among Caribbean adolescents were also discussed)[23,25,72-74,79,80,83,84].

### Violence and Delinquency

Fourteen papers were located under this theme. There were nine peer-reviewed full-text published papers, two theses, one book, one national report and one abstract. Sub-themes included (1) juvenile delinquency, (2) domestic violence and its impact on the adolescent, (3) injuries at the Accident & Emergency (A&E) Department and hospital, and (4) school violence.

#### Juvenile delinquency

The risk factors contributing to juvenile delinquency and school dropouts included a breakdown in family structure, violence in the home, drug use and abuse, association with gangs and economic factors [85] such as, barriers within the educational system, customs and culture [86].

#### Domestic violence

Pupils whose parents were experiencing violent marital discord showed significantly higher levels of both depression and behavioural problems than those pupils not exposed to domestic violence. In addition, "children witnessing domestic violence exhibited more behavioural problems but less depressive symptomatology than adolescents" [87].

#### Violence and hospital admission

At the A&E Departments, patients under 20 years old accounted for 26% of admissions to the emergency room in Trinidad [88]. A review of the adolescent admissions to hospital in Barbados over a 12-month period revealed that 23% were for trauma, 21% were for abortions and 7% were for drug abuse and overdose [89].

#### Violence among secondary school students

Many students had witnessed violence in the home (45%) and school (79%). Many others had personal experience – either causing harm (29%), experiencing harm themselves (20–34%) or having a family member hurt (60%) or killed (37%) [90-93]. Seventy-eight percent of students indicated that they were worried about their safety in going to and from school. Boys, older students and those with lower socioeconomic status reported higher neighbourhood violence. Boys and students from higher socioeconomic status reported higher levels of school violence [92]. Additional statistics on violence-related activity is provided in Additional file 1[23,25,88,91].

### Eating Disorders and Obesity

The research yielded seven papers: three full-text publications and four other papers available only as abstracts. Papers were focused on two areas of interest: eating disorders and weight control behaviour; and body image, physical activity and obesity.

#### Eating disorders and weight control behaviour

In 1991, anorexia nervosa was found to be more common in the higher socio-economic group and young females seldom choose food refusal as a method of expression of weight controlling behaviour in Barbados [94]. In another study in 2004 although 11% were clinically significant on a screening test, no students were diagnosed with bulimia on the Bulimia Diagnostic Interview (DSM III-R). An increased Body Mass Index (BMI) was associated with being terrified of becoming fat, fat-fear, dieting and exercising to lose weight. The distribution of the screening score was not affected by ethnicity or social class; however, girls of Afro-Caribbean origin expressed more concerns with respect to eating habits. In particular, it was noted that there was a sense of lack of control over food, food dominated their lives, they ate in secret and there was the urge to binge [95]. In another study in 2002, weight-controlling behaviour was prevalent and was found to be similar across genders. This study also showed that while Caribbean adolescents reported lower levels of weight and body dissatisfaction compared to adolescents in the United States, Caribbean adolescents reported higher levels of extreme dieting behaviour such as induced vomiting and taking diet pills. More girls than boys were dissatisfied with their weight and bodies. A higher percentage of girls than boys reported that they dieted or exercised as a method to lose weight. More boys reported they had taken laxatives or diuretics and had used vomiting as a means of losing weight (all significant at *p *< 0.05).

Extreme weight-control behaviour was related to several psychosocial factors. Extreme dieters were more likely to report familial problems, be a below average student, have a history of physical and sexual abuse and have had a previous suicide attempt. They also reported more health compromising behaviour, such as, substance use in the past year. Boys who engaged in extreme dieting behaviour were more likely to report that they had run away in the past year and girls were more likely to report that they were sexually active [96].

#### Body image, physical activity and obesity

Two papers originated in Barbados and two in Trinidad and Tobago [97-100]. Generally these papers documented a lack of regular physical activity (about 15%) and between 4–29% being overweight or obese among adolescents. Twenty percent of females and 8% of males misclassified themselves as normal weight.

Overweight Afro-Trinidadian adolescents were more likely to be satisfied with their body size and, conversely, thin south Indo-Trinidadian adolescents were more likely to be satisfied with their body size. The majority of the sample associated normal body size with good health. However overweight was associated with wealth and 40% associated male overweight and obese silhouettes with happiness [99]. Additional statistics on lifestyle issues are included in Additional file 1[97-100].

## Discussion

This extensive review (1980–2005) has documented the prevalence of risk behaviours, outcomes and protective factors in the Caribbean adolescent. These included substance use, high risk sexual behaviour, STIs and HIV/AIDS, teen pregnancy, violence, mental health, and obesity and eating/image disorders. The findings of this review are supported in the international literature where many of the health risk behaviours identified have also included: behaviour that contributes to unintentional and intentional injuries, tobacco use, alcohol and other drug use, sexual behaviours, unhealthy dietary behaviours and physical inactivity [2]. The challenges faced by adolescents and their subsequent negative outcomes have been of growing interest to Caribbean researchers. This trend is revealed by the increasing number of papers publisher: 44 papers in the five years, 2000–2005 compared to 52 papers in the previous 15 years.

### How does Caribbean findings compare with the international literature?

As pointed out by Blum [10] there are similarities in the prevalence of risk factors among adolescents in the United States and the Caribbean. In 2005, there were high reported levels of lifetime use of alcohol (74%), marijuana (38%) and cocaine (8%) in the US [101] as compared with the average Caribbean data of 52% [12,17,19,20,22,24,25,28], 17% [12,14,15,17-20,24,25,28] and 2% [12,17,19,25,26,28], respectively.

In the US, in terms of sexual behaviour, in 2005, 47% of high school students had had sexual intercourse,14% of high school students had four or more sex partners during their lifetime and 34% of currently sexually active high school students did not use a condom during their last sexual intercourse [101]. This current review provides an average of 38% for adolescents who had ever had sexual activity. Of these, 19% had as many as six lifetime partners and 47% who usually used condoms. This data suggests that Caribbean adolescents are possibly participating in high-risk sexual behaviours similar to their US counterparts. It was found that the factors which were associated with this increased adolescent sexual activity included the absence of a father figure, low educational goals and a lack of parental supervision [22,43,44]. Unique to Caribbean countries is the migration of parents to gain employment to help support their families. As a consequence, the care of children and adolescents is entrusted to their elderly grandparents or relatives who are often unable to cope or give adequate supervision. This migration also leads to a disruption in the traditional roles and responsibilities of the family network. Family and school connectedness were shown to protect against early sexual initiation and the ensuing outcomes, such as, early teenage pregnancy and STIs [29,30,32,37,59,60,62].

The risk factors for teenage pregnancy draw a parallel with that of the international community. The teenage mother in the Caribbean is more likely to have been sexually active before the age of 16, be unmarried, have a mother who herself was a teenage mother and have a disadvantaged socioeconomic background [46,48,102,103]. A study on teenage pregnancy in African American adolescents also identified low self-esteem as a risk factor as was found in Jamaica [49,104]. Teenage pregnancies were associated with an increased risk of medical complication such as operative delivery, prematurity and perinatal mortality in all studies except for a study in Trinidad where obstetrical performance matched that of older mothers [54,57].

Suicide is a leading cause of mortality in Caribbean adolescents [3]. Gender differences between adolescents who attempt suicide and those who complete suicide were similar to that of the international literature [105]. The major risk factors were intra-familial and interpersonal conflict, depression, physical and sexual abuse and antisocial disorders, with substance abuse increasing the likelihood of suicidal attempt or completed suicide [70,73,75,77,81]. These findings are comparable with the trends noted in the US [106]; the Netherlands and New Zealand [107]; and the UK [108].

The development of psychopathology in adolescents in the Caribbean was observed by various authors to be associated with the intra-familial conflict [70,73]. Psychopathology in adolescence tends to progress to adulthood [109] but no studies were found which looked at the factors which may act as predictors of progress into adulthood in the Caribbean. Although the adverse situations faced by adolescents may change with the specific culture, the risk factors for increased risk-taking behaviour appear to be universal.

Also reported are high levels of exposure to and participation in violence. One domain not well documented in the international literature is that of rage. It is defined as 'a sense of 'almost always wanting to hurt another'. Five percent of adolescents reported rage in two Caribbean studies [9,29]. Interestingly, comments on studies done in New Zealand in the 1970s, suggested that antisocial behaviour peaks around 16–17 years old and includes 5% of males who were persistent offenders throughout their lives [110]. This is certainly an area for more extensive study and may represent a high risk group that may benefit from a tailored intervention.

Caribbean studies of eating disorders and body image perception mirror that of the data from the US, UK and Europe where anorexia nervosa is a less common presentation than bulimia nervosa [94]. Even though the level of reported body dissatisfaction was lower than in their US counterparts, dieting behaviours of the Caribbean adolescent were more extreme in nature. These extreme dieting behaviours were associated with psychosocial factors such as a history of physical and sexual abuse and, indeed, may serve as a pointer to other risk or health compromising behaviour [96].

While no ethnic differences were observed in studies of bulimic behaviours among school girls in Trinidad and Barbados, girls of Afro-Caribbean origin appeared to have greater concerns about controlling their weight [95]. This finding suggests that further research needs to be done to elucidate any real dissimilarity between the two ethnic groups. Similar findings are mirrored in the other papers from the region on this topic [98,99]. Obesity is an increasing problem internationally and regionally. A source of concern is an observation that 40% of adolescents associated the male overweight and obese silhouettes with happiness [99]. As many as 1 in 7 did not participate in any physical exercise [97]. If the obesity epidemic is not to overwhelm the health care system in the Caribbean then evidence-based solutions are required to halt this mindset favouring the overweight silhouette. Internationally interventions that engender a change in eating behaviour and exercise activity in adolescents stress the need for supportive physical and family environments [111].

### The impact of family instability

At the general population level, international research has identified extreme economic deprivation, conflict in the family, a family history of behavioural problems and a lack of a protective environment as common risk factors for most adolescent substance abuse, delinquency, pregnancy and dropping out of school. Further, the international literature suggests that strategies incorporating positive youth development and resilience have a greater likelihood of improving the health outcomes of adolescent than risk-reduction alone [2].

Similarly, this review suggests that many of the health risk behaviours in Caribbean adolescents are related to their family of origin, home environments and parent-child relationships. Earlier involvement in sexual activity is reported if there is no parental or adult supervision of the adolescent, and if the female grows up in a single parent home. Identified protective factors against early sexual activity include 'better relationships with parents'.

Further evidence of the importance of the parent-adolescent relationship as a protective factor for many risk behaviours were found for the outcomes of teen pregnancy, attempted suicide, depression and teen violence.

### School connectedness

This review has highlighted that the protective effects of school connectedness and increased religiosity noted in US studies were also applicable in the Caribbean. School connectedness appeared to be the strongest protective factor [10]. This suggests the need for a review and remodelling of school education programmes to include promotion of health. Interventions that address healthy eating and fitness, injury prevention and promotion of mental health have been found by researchers to be most likely to be effective [112].

Multi-system interventions that target risky behaviour, such as, violence, school delinquency, drug use and sexual activity have also been shown in male African-American adolescents to impact on rates of health compromising behaviours [113]. These interventions involve school, community networks as well as parents. Although there was no significant effect seen in females these approaches need to be explored further by researchers and policy makers in the Caribbean.

Another protective factor not actively sought by investigators but considered to be essential in promotion of better health among adolescents is their engagement in the social instances that surround them. This may be why one of the principle objective of the United Nations Children's Fund (UNICEF) is that of involvement of adolescent in the decisions that affect their lives [114].

### Limitations of the study

This paper has assembled various studies done on the Caribbean over a 25 year period. While the data reflects the health behaviour of adolescents in the different Caribbean countries the trends do not occur synchronously and care needs to be taken when comparing the data.

Peer-reviewed published articles, country reports, conference papers, theses, and non-peer reviewed papers have been presented as if they are of similar value. Approximately one-third of the papers included were available as abstracts only and 2 published peer-reviewed papers were unavailable despite efforts to acquire full-text copies. This, therefore, limited the data that could be derived for analysis.

Additionally there was little consistency in naming of variables by authors for many of the terms used e.g. 'lifetime use of tobacco' vs. 'cigarette use in the past month' vs. 'cigarette use in the past year'. We have attempted to clarify as far as possible or present data, especially in the tables, which are uniform.

### Documentation and accessibility of research within the Caribbean

At the documentation level, there have been few conversions from CCMRC/CHRC presentations to peer-reviewed publications despite the fact that acceptance of an oral or poster presentation is based on submission of a full-text version of the paper. Twenty-eight papers published as abstracts, nine theses that were not converted into peer-reviewed publications and five papers available only as local reports were identified.

This inability to complete the publication cycle may be as a result of the writing skills of researchers; their confidence in the material; the quality and the analysis of the core data; or may reflect on the large workloads of physicians who do research as a part-time interest and are unable to devote the resources necessary for sometimes numerous and time-consuming revisions.

One problem identified is the difficulty in obtaining papers which have been published on the Caribbean. Often acquisition is only through costly payments to international publishing houses thus making access to information for the average Caribbean researcher very challenging. Sometimes research on the Caribbean may be rejected by international journals as the material may not appear to have worldwide appeal, however, this category of research would be highly relevant to regional researchers. It is felt strongly that systems should be implemented to make valuable research on the region accessible. One suggestion is that a repository be developed for data and papers created regionally.

### Where do we go from here?

It is hoped that future researchers of Caribbean anthropology, sociology and medicine, including adolescent medicine and public health, can view this comprehensive review as a valuable resource. It was found that past and current studies have adequately described risk and protective factors.

In the future, research must begin to investigate the role of interventions at the level of the school, family of origin and the caretaker-child relationship. This may require cohort trials and randomized controlled trials to determine what types of interventions work in our Caribbean milieu. Recent reports coming from Jamaica suggest that a strong Public Health policy with before and after studies can give small countries a powerful indication as to the success of programs [93]. Also, interventional type studies in the Caribbean, especially in the face of the HIV/AIDS epidemic are being undertaken. Five studies which addressed education around sexual issues [115-119]; four studies on school interventions and a fifth on family interventions focussing on safe sexual activity were found. However, these education-based, rather than resilience-based, interventions have been found to be poorly effective, especially over periods greater than 12 months.

There is also a growing presence of educational, social and vocational based programmes in the Caribbean including early childhood education, free tertiary education, youth training programmes and programmes linked to environmental conservation and care of the elderly. Earlier the programme for adolescent mothers to ensure they completed their education was described [59]. These may prove to be inadequate unless we also begin addressing issues around poverty alleviation and a more equitable distribution of wealth, having zero tolerance to violence in the home and in society, supporting single parents, young persons and their families so that they can adequately provide a loving and nurturing environment to develop adolescents and youth with high personal resilience and self-esteem.

As noted in the discussion above there is need for interventions to support the family to carry out its primary role. The future will tell if these programmes have an impact on the high prevalence rates recorded in the last 25 years and documented in these pages.

## Competing interests

The authors declare that they have no competing interests.

## Authors' contributions

All authors contributed equally.

## Supplementary Material

Additional file 1 **Table S1.** Summary Table of papers on Adolescent Health in the Caribbean. Click here for file
