# Supplementary material for: Health risk behaviours among adolescents in the English-speaking Caribbean: a review
Source: Child Adolesc Psychiatry Ment Health. 2009 Mar 17;3:10. doi: 10.1186/1753-2000-3-10 (PMC2667478; doi:10.1186/1753-2000-3-10)
Supplement: Additional file 1 — Table S1. Summary Table of papers on Adolescent Health in the Caribbean. [file 1753-2000-3-10-S1.doc]

## Table S1 - Summary Table of papers on Adolescent Health in the Caribbean

| **No.** | **Reference No.** | **Citation** | **Sample Description** | **Methodology** | **Results** | **Associated Risk / Preventive factors identified** |
| --- | --- | --- | --- | --- | --- | --- |
| 1 | 6 | Singh H, Mustapha N: **Some factors associated with substance abuse among secondary school students in Trinidad and Tobago.** *J Drug Educ* 1994, **24**(1):83-93. | 1603 secondary school students aged 14-18 in Trinidad and Tobago | Multi staged sampling with stratification of island into health administrative areas and selection of secondary schools with probability proportionate to the size of population and selection of seniors. Self-administered questionnaire completed in classroom and supervised by trained supervisor.  80% response rate. | Students at all levels of academic performance were involved in substance use. | Students at lower levels of academic performance were more likely to be involved with substance use.  Adolescents who perceived religious youth programs as important, believed in the importance of prayer, or had a strong perception of the importance of religious counsel or teaching, tended to be less involved in substance use when compared with those who perceived these factors as unimportant.  Students who received higher levels of spending money were more likely to be involved in substance use.  Students whose fathers or mothers used alcohol were more likely to admit to using alcohol when compared with children of parents who did not use alcohol.  Students who were likely to perceive themselves as ‘bright’ or ‘likely to come at the top of the class’ were less likely to be involved in substance use. |
| 2 | 7 | Boyd-Patrick HA, Forsythe-Duke V, Edwards R, Holder Y: **Behavioural risk factors in the adolescent and adult populations of Trinidad and Tobago, 1989 [abstract]**. *West Indian Med J* 1992, **41**(Suppl. 1):16. | 306 adolescents (16-19 yrs old) | 2-stage sample of 1,700 households.  1,448 (36% of target sample) persons to study non communicable diseases. Participants were interviewed with a KAPB questionnaire. | 4.8% of adolescent males and 14.9% of adult males were former or current smokers (100 cigarettes per lifetime).  Smoking started before 25 yrs old.  One in 2 of all smokers had attempted to stop but was susceptible to advertisement. | Not stated. |
| 3 | 8 | Harvey SC: **Patterns of drug abuse in persons referred to the drug rehabilitation unit in Barbados [abstract].** *West Indian Med J* 1997 Apr, **46**(Suppl. 2):39. | 197 cases analysed- 182 males and 15 females  Mean age =31  Range 14 – 69 yrs old | A review of case notes | 53.6% were referrals from the Psychiatric Hospital; 16.2% self referrals.  Polydrug abuse most common – 34%;  Then rank order: cannabis, alcohol and cocaine.  Most common polydrug was combination of alcohol, cannabis and cocaine – 67%  Most common for adolescents – cannabis at 71.1%, declining to 3.8% in the over 44 yr old group.  Most common for over 44 yrs – alcohol 69.2%, declining to 2.6% in the 16-21 yr old group  27-32 yr olds had the greatest use of cocaine | Use of cannabis as a risk factor for adolescents to use cocaine during productive, adult years.  Concern about polydrug use. |
| 4 | 9 | Blum RW, Ireland M: **Reducing risk, increasing protective factors: findings from the Caribbean Youth Health Survey**. *J Adolesc Health* 2004 Dec, **35**(6):493-500. | Caribbean Youth Health Survey (n= 15 695). African 78.5%, East Indian 6.5%, Amerindian 5.5% descent. | Data from an anonymous survey of 9 countries in Anglophone Caribbean using a pre-tested 87-item pencil and paper instrument derived from the Minnesota Adolescent Health Survey and the Youth Risk Behavior Survey.  They also analysed the results of the *Caribbean Youth Health Survey* studying the prevalence of health-compromising behaviours. The four health-compromising behaviours studied were tobacco use and alcohol use, sexual intercourse and involvement in violence. They also attempted to determine the risk and protective behaviours associated with these for the same population. | Rage, defined by the authors as “thinking of hurting or killing someone” was the strongest risk factor for every health-compromising behaviour for both genders and across all age groups. 6% males and 4% of females reported rage. When “reported rage, skipping school and abuse” were reported violence occurred in 91% of males and 77% of females versus 22% and 8% for each gender, respectively, when none of the risk factors were present. Protective factors were considered to be parental and school connectedness. Parental connectedness was determined by the responses to items such as: feel parents care, can tell parents about their problems, feels other family members care, feels people in the family understand and family pays attention to you. It was found to be associated with a lower likelihood of reporting poor or fair health, and protective of suicide attempts in all age groups. Among those aged 16 and below parental connectedness was protective against violence and promoted a delay of sexual activity.  School connectedness based on the questions “do you get along with teachers and do you like school” was observed to be the strongest protective factor. Family connectedness and religious attendance had roughly equivalent protective association. Risk factors as well as protective factors were shown to be cumulative for the four health-compromising behaviours studied by Blum and Ireland. They concluded that for many of the outcomes increased protective factors were associated with as much or more reduction of involvement in health-compromising behaviours than a decrease in risk factors. | Rage was defined by the question ‘Do you ever think about hurting or killing someone?’ Rage was the strongest risk factor for violence involvement, sexual intercourse, tobacco use and alcohol use, across all age groups and both genders.  School connectedness was the strongest protective factor. |
| 5 | 10 | Blum RW, Halcón L, Beuhring T, Pate E, Campell-Forrester S, Venema A: **Adolescent health in the Caribbean: risk and protective factors.** *Am J Public Health* 2003 Mar, **93**(3):456-460. | Caribbean Youth Health Survey (n= 15 695). African 78.5%, East Indian 6.5%, Amerindian 5.5% descent. | Data from an anonymous survey of 9 countries in Anglophone Caribbean using a pre-tested 87-item pencil and paper instrument derived from the Minnesota Adolescent Health Survey and the Youth Risk Behavior Survey. |  | Across all age groups, both parental substance use and parental mental health problems were associated with substance use. In most age groups studied, rage, abuse, parental violence and a friend or family member who attempted suicide were found to correlate highly with substance use.  Across all age groups, the strongest association with attempted suicide was a history of a friend or family member’ suicide.  Parental connectedness was strongly protective against suicide in all age groups.  23% of those who experienced sexual abuse reported attempting suicide vs. 9.1% of persons not experiencing abuse reporting attempted suicide.  Connectedness to parents was strongly protective with delayed sexual initiation among those less than 16 yr.  Both rage and physical or sexual abuse were associated 6with early sexual intercourse among all age groups. |
| 6 | 11 | Ohene S, Ireland M, Blum R. **The clustering of risk behaviors among Caribbean youth**. Matern Child Health J. 2005;9(1):91-100 | Caribbean Youth Health Survey (n= 15 695). African 78.5%, East Indian 6.5%, Amerindian 5.5% descent. | Studied the associations between cigarette smoking, alcohol and marijuana use, early initiation of sexual intercourse, involvement in violence and delinquency using odds ratios and also used survival analysis to determine the association between the initiation of sexual activity and risk behaviours. Data from an anonymous survey of 9 countries in Anglophone Caribbean using a pre-tested 87-item pencil and paper instrument derived from the Minnesota Adolescent Health Survey and the Youth Risk Behavior Survey. | Percentages of school-going adolescents reporting risk behaviours in the presence of 3 protective factors: school connectedness, family connectedness and religious attendancePercentages of school-going adolescents reporting risk-behaviours in the presence of 3 risk factors: abuse, skipping school and rage.  | **Risk Behaviours** | **Males (%)** | **Females (%)** | | --- | --- | --- | | Violence | 27 | 6 | | Sexual activity | 34 | 7 | | Alcohol use | 3 | 1 | | Tobacco use | 5 | 2 |  | **Risk Behaviours** | **Males (%)** | **Females (%)** | | --- | --- | --- | | Violence | 91 | 77 | | Sexual activity | 88 | 74 | | Alcohol use | 44 | 54 | | Tobacco use | 50 | 37 | | Consistently relationships were strongest for females and substance use e.g. OR=44.4 for cigarettes and marijuana and OR=48.2 for alcohol and cigarette combinations; as compared to 16.1 and 22.1 for male for the above combinations.  The association between weapon-related violence and gang involvement was higher for males OR=6.7 vs. females OR=5.5.  Early sexual involvement among the 10-12 year-olds was significantly correlated with alcohol use, gang involvement, weapon-related violence, running away and skipping school (OR= 1.7-3.6 and *p* < 0.0001 for each risk behaviour).  Among the 13-15 year olds the correlations between the risk behaviours were stronger for females than males: The odds that a female who smoked cigarettes also used marijuana was 50.7 vs. males OR= 15.8.  An important and potentially troubling aspect of the risk behaviours noted among Caribbean youth is the aspect of the ‘clustering’ of these behaviours. Ohene and colleagues in a secondary analysis of the *Caribbean Youth Health Survey* studied the associations between cigarette smoking, alcohol and marijuana use, early initiation of sexual intercourse, involvement in violence and delinquency using odds ratios and also used survival analysis to determine the association between the initiations of sexual activity and risk behaviours. They reported that relationships were consistently strongest for females (if a female is involved in one risk behaviours there is a strong association with being involved in another) and were most notable for the substance use variables and conclude that future program developers should not focus only on specific behaviour but should aim for interventions that cover the breadth of risk behaviours. |
| 7 | 12 | Smart RG, Patterson SD: **Comparison of alcohol, tobacco, and illicit drug use among students and delinquents in the Bahamas**. In: *Drug Abuse Scientific Publication 522.* Washington D.C.: PAHO; 1990. | 4 767 students in 32 schools and from 192 classes.  74 delinquents. | Self-administered questionnaire among schools and staff administered at delinquents’ institution. | Among students: Alcohol use (66%); tobacco (14.7%); marijuana (8.2%); cocaine (1.7%).  Among delinquents:  Alcohol use (74%); tobacco (32%); marijuana (57%); cocaine (11%); Tranquillizers (15%). | Marijuana users at junior and senior high schools were more likely male, older 17 &19, lived with 1 parent, had lower grades at school, les often expect to graduate or attend college, not religiously involved, father unemployed, employed in hotel industry, or sells drugs,  Mother sells drugs, family member have used marijuana, father drinks alcohol regularly.  Marijuana users at delinquents’ institution were male, formerly lived with fathers, had more spending money, not religiously involved, fathers in business, security work or professions, mother sold drugs, worked in offices, or no mother, families used or sold drugs. |
| 8 | 13 | Soyibo K: **Use of alcohol, tobacco and non-prescription drugs among Jamaican high school students.** *West Indian Med J* 1997 Dec, **46**(4):111-114. | 2,417 Jamaican high school students (1,063 boys, 1,354 girls). | Pre-tested questionnaire administered under examination conditions by class teachers. | Test-retest correlation of 0.95.  The prevalence of alcohol and tobacco use was 50.2% and 16.6%, respectively. | Females were more likely to use paracetamol, aspirin or multivitamins.  Alcohol and tobacco use was higher among males, urban students and children of professionals when compared with females, rural students or children of non-professionals. |
| 9 | 14 | Thompson SA, Paul TJ, Holder-Nevins D: **To smoke or not to smoke: understanding ganja use in adolescents [abstract]**. *West Indian Med J* 2005, **53**(Suppl. 2):35. | 210 students aged 12 – 17 from high schools in St. Thomas, Jamaica | Self-administered questionnaire to determine the effect of attitude and sub-adolescents in students were randomly selected. 18 students participated in focus groups. | One third of students had smoked ganja (37.5 % boys , 24.8% girls) | Having friends who supported ganja smoking.  A household who supported ganja smoking. |
| 10 | 15 | Figueroa JP, Ward E, Walters C, Ashley DE, Wilks RJ: **High risk health behaviours among adult Jamaicans**. *West Indian Med J* 2005 Jan, **54**(1):70-76. | 168 adolescents aged 15-19 years | A nationally representative random sample in 56 enumeration district from which 2013 persons were drawn. Survey using pre-tested questionnaire along with basic anthropometrics and BP. | Among 15-19 year olds: Current smokers: 10.8% males & 1.1% females  Ever smoked marijuana: 31.5% males & 7.4% females  Ever had an STD: 3.6% males & 6.5% females  Multiple sex partners in the last year: 47.6% M & 20.4% F  Condom use at last sex for those with more than 2 partners in the last year: 70% M & 55.6% F | Not stated. |
| 11 | 16 | Boyd-Patrick HA, Forsythe-Duke V, Edwards R, Holder Y: **Alcohol drinking practices in the adolescent and adult population in Trinidad and Tobago, 1989 [abstract].** *Caribb Med J* 1992 Sep, **53**(Suppl. 1):9. | 1,448 adolescents from 11 schools and 1700 households | Pertinent interview and Questionnaire – a KAPB Study | 42.5% of adolescent males and 46.1 % of adult male, 11.3 % of adolescent females and 21.9% of adult females drank at least once a month.  Acute heavy drinking (5 or more drinks on a single occasion) and solitary drinking were noted in female adolescents.  The CAGE questionnaire revealed alcohol abuse patterns in 2.9% of adolescent.  Male pattern of alcohol consumption are similar in adolescents and adults suggesting early habit formation.  6.9% of young males have already demonstrated an additive pattern of drinking. | Health prevention programmes need to be initiated in schools to prevent alcohol abuse  The dangers of alcohol excesses need to be highlighted e.g. drinking and driving.  The availability of alcohol to minors and the promotion of alcohol by advertising and screening of drivers for alcohol levels and other preventative measures need to be addressed. |
| 12 | 17 | Singh H, Maharaj HD, Shipp M: **Pattern of substance abuse among secondary school students in Trinidad and Tobago.** *Public Health* 1991 Nov, **105**(6):435-441. | 1603 secondary school students aged 14-18. | Multi staged sampling with stratification of island (Trinidad and Tobago) into health administrative areas and selection of secondary schools with probability proportionate to the size of population and selection of seniors. Self-administered questionnaire completed in classroom and supervised by trained supervisor.  80% response rate. | Lifetime use of alcohol 84%; tobacco 35%; marijuana 8%; cocaine 2%; both alcohol and tobacco 20% both tobacco and marijuana 16.8%; both cocaine and marijuana 1.8%.  Use of alcohol in last month 22%.  46% were provided with their first drink of alcohol through a family member or parent. | Indo-Trinidadians as compared with Afro-Trinidadians were more likely to have used alcohol in the last month..  Students whose fathers used alcohol were more likely to admit to using alcohol when compared with children of fathers who did not use alcohol.  Afro-Trinidadians were more likely than Indo-Trinidadians to use marijuana. |
| 13 | 18 | Douglas KG: **Patterns of substance use among post primary students in Jamaica, 1997: prevalence and long-term trends [abstract]**. *West Indian Med J* 1999, **48**(Suppl. 1):24. | 7996 students (grades 9, 11 and 13) | Study on prevalence of alcohol, cigarettes, and other drugs use by adolescent school population and to describe the long-term trend of drug use comparing 1987-1997 data. | Between 19987 and 1997:  Lifetime prevalence of smoking cigarettes decreased form 29.1 to 27.2 %; alcohol use decreased (76.3-70.9% but remained high; marijuana use increased from 19.8 to 26.9%.  Annual prevalence for marijuana increased by 8.9% and current prevalence by 3.6%.  Prevalent use of inhalants remained high at 15.8% lifetime.  Among current uses, alcohol had the highest prevalence – 28.8%, inhalants – 9.6%, marijuana tea – 6.1% and cigarettes – 4.8%.  Compared to 1987, there were significant decreases in current prevalence of use of alcohol (p<0.001) and non-prescribed amphetamines (p<0.05) but smoking of marijuana increased by 3.6%.  Thirty-day prevalence of use among males and females showed significant increases or marijuana and non-prescribed tranquilizers (male only) and both showed significant decrease in use of alcohol.  For 9^th^ graders – smoking marijuana and use o inhalants increased, while use of alcohol decreased.  For 11^th^ graders – smoking marijuana increased significantly but use of alcohol and inhalants decreased.  Use of non-prescription tranquillizers showed significant increase. Frequency of use in the previous 30 days (up to two times a week) among males showed a decrease for cigarette smoking but, for both males and females, alcohol use and smoking marijuana increased significantly.  Marijuana smoking was the most likely to be discontinued, followed by use of crack, inhalants and alcohol.  However, in 1987, the discontinuation rate for use of marijuana as tea and smoked was higher.  The incidence use of both alcohol and cigarettes before 7^th^ grade almost parallels the incidence use at 7^th^ grade or after. | High prevalence of cigarette, alcohol and marijuana use is disturbing because of their potential as gateway drug. |
| 14 | 19 | Smikle MF, Dowe G, Hylton-Kong T, Williams E, Baum M: **Risky behaviour in Jamaican adolescent patients attending a sexually transmitted disease clinic.** *West Indian Med J* 2000 Dec, **49**(4):327-330. | 165 consecutive patients (62 males 103 females) attending the Comprehensive Health Centre in Kingston | Pre-tested structured questionnaire, clinical history, examination, blood samples of adolescents conducted in 1997-8 | 4% reported using condoms consistently and 36% never used condoms.  36% used marijuana, 10% used alcohol and 1% used cocaine.  33% had repeat infection and 1.2% had co-infection with HIV | Mean age of first sexual contact was 14.6 +/-1.5 years and 12.5+/-2.5 years for females and males respectively.  Males tended to have more sexual partners OR=7.0 (95%CI, 2.9-17).  More males than females used marijuana (OR 3.0 (95% CI 1.9-4.1)) or alcohol (OR 2.3 (95%CI 1.8-3.5)).  Females were more likely than males to have had a previous STD (OR 2.3 (95%CI 1.0-1.7)), or to admit that their sexual partner used marijuana.  After adjusting for gender, marijuana smoking was significantly associated with the presence of dysuria (OR 2.0 (95%CI 1.6-3.4)). |
| 15 | 20 | Ivey MA, Douglas KG, Casimir LB, Prince PE: **Substance use in Caribbean secondary schools, the Commonwealth of Dominica and St. Kitts and Nevis experience: preliminary results to a regional perspective [abstract]**. *West Indian Med J* 2003 Jun, **52**(Suppl. 3):53. | 4467 students – 57% in Dominica  Mean age of 13, 15 and 17 for 2^nd^, 4^th^, and 6^th^ form students | Survey of secondary school in Dominica and St. Kitts and Nevis  Conducted with self-administered questionnaire in the classroom | Lifetime prevalence  72% - alcohol  22% - cigarettes  17%- marijuana  3% - tranquilizers  3%- solvents  0.5% - cocaine  Current prevalence  28% - alcohol  3% - cigarettes  4%- marijuana  Lifetime use more prevalent for males than females:  78% vs. 70% - alcohol  31% vs. 16% - cigarettes  23% vs. 12%- marijuana  11% and 18% of students perceived frequent use of marijuana and alcohol to be slightly or not so harmful.  20% were curious about trying an illicit drug and 15% said yes they would try given the opportunity and 7% said they would definitely try one.  Alcohol is the legal substance of choice.  Marijuana is the illegal substance most used.  59% had not taken any prevention programmes. | Not stated. |
| 16 | 21 | Antoine AA: **Knowledge, attitude and behaviour regarding tobacco use and environmental tobacco smoke among Grenadian students [abstract].** *West Indian Med J* 2004, **53**(Suppl. 2):42. | 3428 primary and secondary school students  (1770 girls and 1342 boys) | Schools selected in a 2-stage cluster design; Cross-sectional survey using self-administered questionnaire in a classroom setting between June and July 2000 to examine Grenadian students’ attitude, knowledge and behaviour related to tobacco use and environmental tobacco smoke | Response rate – 73.3%  28.0% having tried at least one cigarette  Current use of any tobacco product 16.4%  Current use of cigarettes- 9.0% with 0.5% smoking frequently  Students smoked  At home( 32% girls, 49% boys)  Friend’s house – 17.5%  Think that smokers have more friends – 27% boys and 17. % girls.  Think smokers more attractive – 9% boys and 8.2% girls  70% think second-hand smoke harmful  72.1% think smoking should be banned in public places  Exposed to tobacco smoke –  30% at home and 53.7% outside home  About 33.3% have one or more parents who smoke.  Tobacco use low in Grenada  Exposure to ETS is moderately high | Not stated. |
| 17 | 22 | Kurtz S, Douglas KG, Lugo Y: **Sexual risks and concerns about AIDS among adolescents in Anguilla**. *AIDS Care* 2005, **17**(Suppl. 1):36-44. | 1,225 enrolled school students ages 10-20. | Survey using a self-administered questionnaire as part of national Adolescent Health Survey between March and April 2002. | 43.3% males and females admitted to alcohol use in the past 12 mo. And 10.4% males and 6 % females admitted to marijuana use. 28.3%males and 14.8% females had experienced sexual intercourse, of these, 78% males and 44.8% females had 2 or more lifetime partners | Predictors of ever having sexual intercourse were being male, alcohol, drug or any substance use in the past 12 months, ever having been physically or sexually abused, feeling depressed in the past month, or past attempted suicide.  Respondents 13 y and older were nine times more likely to use a condom.  Top 3 reasons (of 10) for abstaining from sexual activity included wanting to wait until older (38.2% males and 56.1% females); no opportunity with someone I like (11.7% males and 18.5% females); not being emotionally ready (12.5% males and 13.8% females) |
| 18 | 23 | Halcón L, Blum RW, Beuhring T, Pate E, Campbell-Forrester S, Venema A: **Adolescent health in the Caribbean: a regional portrait.** *Am J Public Health* 2003 Nov, **93**(11):1851-1857. | Caribbean Youth Health Survey (n= 15 695). African 78.5%, East Indian 6.5%, Amerindian 5.5% descent. | Data from an anonymous survey of 9 countries in Anglophone Caribbean using a pre-tested 87-item pencil and paper instrument derived from the Minnesota Adolescent Health Survey and the Youth Risk Behavior Survey. | In general see self as sad, angry or irritable – 16.6%  Ever been physically abused – 15.9% (17% male and 15% females)  Ever been sexually abused – 9.9% (9% males and 11% females)  Ever tried to kill self – 12.1% (11% males and 13% females)  Carried a weapon to school in last 30 days – 12.2% (20% males and 7% females)  Almost always thinking about hurting/killing someone – 4.9%  Ever had sexual intercourse - 34.1%  Age of first intercourse under 10 yrs -42.8%  More than or as many as 6 sexual partners in total – 23.9% | Not stated. |
| 19 | 24 | Douglas KG, Fountain T. **Findings from drug use surveys of Caribbean students, 2002-2003**. West Indian Med J 2004;53(Suppl. 4):52-62 | Approximately 20 000 from grades 8, 10 and 12. | A cross-sectional design of randomly selected students from 10 participating countries using self-administered questionnaires based on surveys. A two-stage sampling process was used to obtain a representative sample of classes. | A response rate of 73.1% was achieved.  Cigarettes was used by 15.3%,  Alcoholic drinks (34.5%), marijuana (6.2%), cocaine (1.2%).  22.3% had experienced an accident or injury as a result of drinking and 13.8% believed that they need help for their alcohol problem. 26.7% had had a completed sexual experience.  13.8% had had sex with 6 or more persons in their lifetime. 7.3% had ever been sexually abused, (4% males and 9% females). Ever experienced physical abuse: 13% males and 17% females).  11% saw themselves as sad, angry or irritable, and 5% expressed the desire to ‘always wanting to hurt someone’. 11% male and 4% females admitted to carrying a weapon to school in the last 30 days | Not stated. |
| 20 | 25 | Ministry of Health, Trinidad and Tobago. Pan American Health Organization: **Adolescent Health Survey**. Port-of-Spain, Trinidad: Ministry of Health; 1998. | 878 adolescents 10-20 years from throughout the school system in Trinidad and Tobago. | A self-administered anonymous questionnaire. | Cigarettes was used by 15.3%,  Alcoholic drinks (34.5%), marijuana (6.2%), cocaine (1.2%).  22.3% had experienced an accident or injury as a result of drinking and 13.8% believed that they need help for their alcohol problem. 26.7% had had a completed sexual experience.  13.8% had had sex with 6 or more persons in their lifetime. 7.3% had ever been sexually abused, (4% males and 9% females). Ever experienced physical abuse: 13% males and 17% females).  11% saw themselves as sad, angry or irritable, and 5% expressed the desire to ‘always wanting to hurt someone’. 11% male and 4% females admitted to carrying a weapon to school in the last 30 days. | Not stated. |
| 21 | 26 | Gordon F: **Drug abuse in all age schools**. *Thesis.* Kingston: The University of the West Indies; 1995. | 200 students 13-15yr. | Self administered questionnaire applied to a random sample of students | |  | EVER  % | Last 30 days % | | --- | --- | --- | | Cannabis smoke | 20.3 | 8.6 | | Cannabis Tea | 14.7 | 8.6 | | Cocaine | 3.6 | 3.0 | | Crack | 3.0 | 2.5 | | Tobacco | 5.6 | 2.3 | | Not stated. |
| 22 | 27 | Sharma KLD: **Present trends in drug use and abuse in new secondary schools in the Kingston Metropolitan Area**. *MPH Thesis.* Kingston: The University of the West Indies; 1995. | 200 students aged 15-18. | Survey using stratified sample frame of schools and a random sample of students. | |  | EVER  % | Last 30 days % | | --- | --- | --- | | Cannabis smoke | 39.5 | 23 | | Cannabis Tea | 29.5 | 20 | | Cocaine | 6.5 | 5.5 | | Crack | 6.0 | 3.0 | | Tobacco | 39.5 | 28.5 | | Not stated. |
| 23 | 28 | South West Regional Health Authority: **Healthy communities’ initiative: predisposition to substance abuse among youth 15-24 years old in the South West Region**. Trinidad and Tobago: South West Regional Health Authority; 1995. | 782 youth aged 15-24 years | Survey using a street-intercept method. | 10.7% admitted to marijuana use; 3% to cocaine use; 71.5% to cigarette use; between 16-38.5% to some form of alcohol use. | Not stated. |
| 24 | 29 | Williams YC, Walker SP: **Factors influencing sexual behaviour of 14-15 year olds in Kingston, Jamaica [abstract]**. *West Indian Med J* 2004, **53**(Suppl. 2):19. | 874 grade 9 students in 30 secondary schools in Kingston, Jamaica. | Proportional random sampling used to select students. Self-administered questionnaires to determine the factors influencing early initiation of sexual activity. Case control design used to define cases of sexually-active adolescents | 73% boys and 23% girls had sex. | For boys and older girls:   - Ganja use - Greater alcohol consumption - Involvement in violence   Ganja users were less likely to use condoms.  For boys and girls   - Better relationships with parents decreased risk   Those   - attending church - extracurricular activities - had attended talks on HIV were more likely to use condoms |
| 25 | 30 | Stallworth J, Roofe M, Clark LF, Ehiri JE, Mukherjee S, Person S, Jolly PE: **Predictors of sexual involvement among adolescents in rural Jamaica**. *Int J Adolesc Med Health* 2004 Apr-Jun, **16**(2):165-178. | 788 students 13-19 yrs old. | Cross-sectional survey in Hanover parish, Jamaica. | 62% - had had sex; 38% had not  Mean age of sexual debut 13.6%  For sexual activity –  - being older,  -being male  -having grown-up values | Delay values (Values towards delay of sexual activity) – protective  Higher self –efficacy skills was predictive of adolescent sexual involvement  By gender – delay and grown-up values predicted male sexual activity  -self-efficacy, paternal love and delay values predicted female behaviour  - need for gender-specific interventions |
| 26 | 31 | Dzakpasu-Lawrence PP: **Knowledge, attitudes and practices regarding syphilis and gonorrhoea among fourth form secondary school students in Kingston, Jamaica**. *MPH Thesis.* Kingston: The University of the West Indies; 1996 | 194 4^th^ form students 13-17 yrs in Kingston, Jamaica. | Four schools participated using a pre-tested questionnaire  96 received formal health education  99 no formal health education | 51.9% females had had sex  75.5% males had had sex  38.3% used condoms always | Not stated. |
| 27 | 32 | Ohene SA, Ireland M, Blum RW: **Sexually-inexperienced Caribbean youth correlates of delayed sexual debut**. *Adolescent & Family Health* 2004, **3**(4):177-184. | 8,784 sexually inexperienced youth aged 10-18 from Caribbean Youth Health Survey | Data from an anonymous survey of 9 countries in Anglophone Caribbean using a pre-tested 87-item pencil and paper instrument derived from the Minnesota Adolescent Health Survey and the Youth Risk Behavior Survey. | Sexual inexperience decreased with age.  Across all age groups girls were more likely to report not having had intercourse than males. 71.3% males and 91.0% females aged 10-12 admitted to never having sexual intercourse. 26.8% males and 53.6% females aged 16-18 admitted to never having sexual intercourse. | HIGHLIGHTS OF THE FINDINGS INCLUDE:  Males and female who liked school were less likely to report fear or concerns about consequences of sexual activities as the reason for delayed coitus.  Males and female who saw themselves as religious were more likely to ‘wait until married or older’ to initiate coitus.  Males and females who reported liking school, attended religious services (except for girls aged 10-12), experienced family connectedness, and had married parents were significantly less likely to cite the ‘lack of opportunity to have sex’ as an explanation for not being sexually active.  For males selecting ‘not being ready to have sex’ was associated with attendance at religious services. |
| 28 | 33 | Eggleston E, Jackson J, Hardee K: **Sexual attitudes and behaviour among young adolescents in Jamaica**. *Int Fam Plan Perspect* 1999, **25**(2):78-84, 91. | 945 students aged 11-14 | Data from a survey and information from a set of focus group discussions. | Mean age at first intercourse was 9.4y and 11.3y for males and females respectively.  Only about one third of both males an females were unaware that pregnancy was possible at first intercourse | 64.4% males and 5.8% females reported that they had experienced sexual intercourse.  Mean age difference between first partner’s age and respondent’s age was 3.2y for females and 1.2y for males.  77.7% males vs. 52.5% females agreed that condoms protected against STDs.  30.3% males vs. 14.9% females agreed that having sex while standing prevents pregnancy.  71.4% males vs. 53.9% females agreed that condoms were only for boys who have sex with more than one girl. |
| 29 | 34 | Waldrond ER, Hoyos MD, Souder M, Jones F, Ellis H, Roach T: **Sexual attitudes and practices amongst schoolchildren aged 10 -16 years in Barbados [abstract].** *West Indian Med J* 1991, **40**(Suppl. 1):23. | 9% systematic sample of all school children in Barbados | Self-administered questionnaire /knowledge, practices and attitudes survey on knowledge about AIDS. | Basic knowledge – 92%  Knowing that it was sexually transmitted – 89.5%: 27.8% of children thought that they should have sex before marriage or settle with one partner; 51.4% boys and 18.5% girls were sexually active; 66.1% of the sexually active had 2 or more partners and this % fell to 25.5% in the past year. ¾ of the sexually active children knew how to use condoms, boys 320 (78%) claimed more skills than girls 127 (68%). | Sexual attitudes and practices among schoolchildren are important in the AIDS epidemic as many of the AIDS patients are in the 19-26 yr age range.  With a 10 yr symptomatic period between initial infection and clinical manifestation, the virus probably acquired in their school years. |
| 30 | 35 | Kumar A, Carter R, Doughlin C, Kumari G, Jacob C: **Risk behaviour and predisposition to HIV infection among the secondary school students in Barbados - results from a national survey [abstract]**. *West Indian Med J* 2005, **54**(Suppl. 2):22. | 922 youths – ages 11-18 in Barbados. | Cross-sectional survey from all secondary schools.  Multi-stage systematic random sampling.  Self-administered questionnaire.  Measured family and socio-demographic characteristics, sexual history and risk-taking behaviour, drug use, risk perception with respect to HIV infection | Males slight majority of sample  21.6% - vaginal sex  10.2 % - oral sex  2.5 % - anal sex  4.6% had sex <10yrs  29.1% had sex 11-15 yrs old  In last 4 months  37% used condoms consistently  37% never uses condoms  Reasons for non-use  23.1% regular partner  23.1% condoms uncomfortable  20.5% unable to get condoms  67.1% felt confident that they could refuse sex without a condom  Rating own infection rate – 28.1% felt that there was no risk  30.9% felt that risk was low | - Early onset of sex - Limited negotiation skills and power in sexual encounters - Multiple sex partners - Low/inconsistent condom use |
| 31 | 36 | Allen C, Martinez DD, Wagner U, McLetchie K, Washington AD, Chapman-Smith T, Wright M: **The sexual behaviour of youth in Tobago: a report on the development of a health promotion project**. *West Indian Med J* 2002 Sep, **51**(3):197-199. | 676 persons (44% males and 56% females) aged 10-29 across Tobago. | A survey. | Age of first sexual intercourse for males and females was 13.1 and 15.2 respectively. 20% of 10-14 year olds, 25% of 15-19 year olds in school and 36% of those out of school reported more than 5 partners. 64% of the sexually active never used condoms | Males reported significantly higher numbers of partners than females. |
| 32 | 37 | Norman LR: **Sexually transmitted disease symptoms: a comparative analysis of male and female youth in Jamaica**. *West Indian Med J* 2001 Sep, **50**(3):203-208. | 1632 and 1351 sexually experienced males and females respectively in Jamaica. | A 3-stage national probability sample with household based interviewer-administered questionnaire. | Mean age at first intercourse was 13.6 and 15.7 for males and females respectively.  32.7% males and 2.8% females had multiple partners in the last 3 months.  5% males and 1.3% females admitted to having to treat themselves for STI such as gonorrhoea or syphilis. | For every year increase in level of education, the odds of reporting STD symptoms decreased by 0.87.  For every year increase in the age of first intercourse, the odds of reporting STD symptoms decreased by 0.92.  Males expressing cultural attitudes supporting high risk behaviours were more likely (OR 1.54) to report symptoms of STD than those with no such attitudes.  Males who reported having multiple sex partners were more likely (OR 2.69) to report STD symptoms. Males who reported consistent condom use with steady partners were less likely (OR 0.58) to report symptoms of STD than were inconsistent users. |
| 33 | 38 | Jagdeo TP: **Myths, misperceptions, mistakes: A study of Trinidadian adolescents**. Port-of-Spain: Family Planning Association; 1986. | 398 adolescents aged 15-22. | Starting from a random point in in 4 areas-Laventille, Marabella, Charlieville and Valencia, every third household was contacted and a list of all males and females aged 15-22 was taken. Interviewers then used a random table printed on each questionnaire to identify the person to be interviewed. Respondents were only interviewed by members of their same ethnic group and gender. | Respondents of African descent were almost twice as likely to have started sexual activity (58.9% vs. 32.6%). Males were more likely to report sexual activity (61.5%vs. 42%). 23.5% of those aged 15 and 50% of those aged 17 reported sexual activity. | 56% of respondent placed the ‘blame’ for sexual activity on peer patterns. |
| 34 | 39 | Knight RC: **A knowledge, attitude and practice study with respect to family planning among high school students in Barbados and Jamaica**. *Thesis.* Kingston: The University of the West Indies; 1989. | 153 Barbadians and 153 Jamaican; 12-14 year attending old co-ed schools | Cross-sectional; study using a pre-tested questionnaire | 42.5% Barbadian reported Sexually Active  22.2% Jamaican reported Sexually Active  14% of Barbadian and 10% of Jamaican males admitted to first intercourse before 10 years of age.  2% of females in both populations admitted such. | Not stated. |
| 35 | 40 | Lerand SJ, Ireland M, Blum RW: **Sexual behavior in Caribbean youth**. *Journal of Adolescent Health* 2004, **34**(2):142-143. | Caribbean Youth Health Survey (n= 15 695). African 78.5%, East Indian 6.5%, Amerindian 5.5% descent. | Data from an anonymous survey of 9 countries in Anglophone Caribbean using a pre-tested 87-item pencil and paper instrument derived from the Minnesota Adolescent Health Survey and the Youth Risk Behavior Survey. | Not stated. | Not stated. |
| 36 | 41 | Smith D, Roofe M, Ehiri J, Campbell-Forrester S, Jolly C, Jolly P: **Sociocultural contexts of adolescent sexual behavior in rural Hanover, Jamaica.** *J Adolesc Health* 2003 Jul, **33**(1):41-48. | 73 out-of-school adolescents 15-18 yrs old in Hanover, Jamaica. | Focus group discussions to investigate sexual behaviour of adolescents. | Existence of different sexual scripts for males and females. | Perception of vulnerability to HIV/AIDS and other STDs and knowledge of sexual risks were low, often erroneous.  Abstinence – more desirable by females who tend to be culturally restrained  Both sexes felt that family life was important and had strong influence on sexual behaviours  Cultural and gender norms impose different standards on the sexes |
| 37 | 42 | Jackson J, Leitch J, Lee A, Eggleston E, Hardee K: **The Jamaican adolescent study - final report.** Research Triangle Park, North Carolina: Women's Studies Project, Family Health International (FHI); July 1998. | 945 students in seventh grade 10 secondary schools in Jamaica.( 50% urban or periurban and 50% rural)  Mean age 12.1 years | Quasi experimental study design with comparison of groups. Multistage sampling using pre-test/post test immediate and 1 year after intervention  Focus group interviews  Intervention and in-school family life education programme. Study between 1994-1996 | 52% female and 48% males  42% lived with mother and 37% both parents  62 % attended church at least once weekly | Logistic regression analyses were used to assess significance of impact on attitudes:  While adolescent attitudes were more supportive of family planning, project was not significant in the short term or the long term  There was a favourable short term impact on pregnancy attitude “*girl should have a baby as teen to prove fertility,* but no long term impact |
| 38 | 43 | Wyatt G, Durvasula RS, Guthrie D, Lefranc E, Forge N: **Correlates of first intercourse among women in Jamaica**. *Arch Sex Behavior* 1999, **28**(2):139-157. | 897 women | Retrospective reports of a random sample of Jamaican women. | Not stated. | Early initiators were more likely to have had less early family stability and to have experienced menarche at a younger age than late initiators. Although early initiators of intercourse were more likely to report lower socioeconomic status, less STD knowledge, and greater numbers of pregnancies, they were no more likely to report more sexual partners than women who engaged in first intercourse after the age of consent, and had a greater number of long-term relationships.  Women growing up in one-parent families were more likely to engage in intercourse before the age of 16. The age of menarche was also significantly associated with age of intercourse. The characteristics of the first sexual partner and the characteristics of the relationship were significantly associated with the age of intercourse. On the other hand, there are four sets of variables assessing current demographic characteristics, current relationship status, and factors such as religiosity and knowledge on sexually transmitted diseases (STDs). |
| 39 | 44 | Bain BC, White B, Madden F, Bain P, Anderson-Johnson P: **Factors in the home environment which influence sexual knowledge and sexual activity among pre-adolescents in Kingston and St. Andrew, Jamaica - implications for prevention of HIV infection [abstract]**. *West Indian Med J* 1993 Nov, **42**(Suppl. 3):27-28. | 518 school children, aged 8 – 12 yrs old in 15 schools | Multi-stage sampling technique  Structured questionnaire re: living arrangements, relationship between parents/guardians, knowledge about sex and STDs, and sexual experiences.  Analysed correlation between individuals. | Higher levels of sexual activity if :  (a) no adult in charge when parents were away (sibling overseeing)  (Relative Risk = 3.98; p<0.001)  (b) not occupied with chores or homework after school (RR= 2.0; p=0.01)  (c) sharing sleeping  facilities (RR = 1:86; p = 0.01)  (d) visit neighbours or friends without requiring parents or guardians permission (RR =2.40, 2.75; p<0.001, 0.0001, )  Travelling patterns of parents did not affect sexual activity of children  31% learnt of sex from TV  12% learnt of sex from parents  86% had heard of AIDS | 56 % wanted parents to teach them about sex  43% wanted parents to teach them about STDs |
| 40 | 45 | Olenick, I. **Among young Jamaicans, sex and childbearing often begin during adolescence** *International Family Planning Perspectives* 1999 Dec, **25**(4):206-7. | 1,191 women and 2,279 men aged 15–24 | Data taken from1997 Jamaica Reproductive Health Survey | Sexual initiation  Average age first intercourse was 13.4 years for males and 15.9years for females  Fertility  The age specific fertility rates for women aged 15–19years was 112 births per 1,000 as compared to 163 per 1,000 women aged 20–24 years | Sexual initiation varied with church attendance: 49% of females who attended church had begun having sex while 83%of those who never attended church had initiated sex; 70 %of males who attended church vs.92% of males who never attended church  The younger a female was at first intercourse, the older her first partner was likely to be (p.206)  Fertility rates were higher for women in rural areas than for those in urban areas  The likelihood of a pregnancy or live birth decreases with increase in level of education and socio-economic status |
| 41 | 46 | Archer EY, Campbell J, Medford G, Scantlebury MA: **Profile of teenage mothers and their parents' attitudes to teenage sexuality and pregnancy [abstract]**. *West Indian Med J* 1990 Apr, **39**(Suppl. 1):17. | 160 teenage mothers (13 – 19 yrs old)  (131 > 16 yrs old (82%) | Questionnaire. | 84% completed secondary schooling  Predominantly of the lower economic class    65% had teenage mothers  71% lived in homes with no male authority  54% had strict parental discipline  59 % had first sexual contact at <16 yrs old  2% were married and 22% were in a common-law relationship  94% had knowledge of contraception before getting pregnant but only 19% practiced family planning  82% had unplanned pregnancies  80% wished to have delayed pregnancies  Sex education – 78% at school and from mother 33% but 78% felt that it was inadequate  Mothers were single parents (71%), of the lower economic bracket and < 40 yrs old  Mothers’ reaction to boyfriend: angry 25%, approving 23%, not angry 15% and no reaction 38%  90% of parents supportive and 2% of teenagers evicted from home | Not stated. |
| 42 | 47 | Harris MI: **Factors that influence the occurrence of teenage pregnancy [abstract].** *West Indian Med J* 2000 Apr, **49**(Suppl. 2):33. | 90 pregnant teenagers, age 14-19 yrs | Case control study of women attending antenatal clinic during Feb and Mar 1995. | Adolescents who became pregnant were 2.7 times more likely to have a mother who was herself a teenage mother.  They were also more likely to have had not discussed human sexuality with parent or guardian | Parenting education, including effective communication skills, is vital. |
| 43 | 48 | Collins-Harris, M: **Factors that influence the occurrence of teenage pregnancies**. *Thesis.* Kingston: The University of the West Indies; 1995. | 45 cases and 45 controls | Case-controlled study |  | 1. More like to have mothers who were themselves teenaged mothers or 2.75 (p=0.02) 2. Live away from their parents or 3.4% (P=0.01) 3. Had no discussion with parents about sexuality or 3.0% (P=0.03) |
| 44 | 49 | Keddie AM: **Psychosocial factors associated with teenage pregnancy in Jamaica**. *Adolescence* 1992, **27**(108):873-890. | 14-17 year olds | Case-controlled study | Father-figure absence and low self-esteem may combine as risk factors for teenage pregnancy in urban Jamaica |  |
| 45 | 50 | Thame M, Wilks R, Matadial L, Forrester TE: **A comparative study of pregnancy outcome in teenage girls and mature women**. *West Indian Med J* 1999, **48**(2):69-72. | 214 teenagers aged 13-19 compared with 297 women aged 22-23 | Retrospective cohort | Not stated. | Offspring of teenagers have lower birth weights (babies of younger mothers were 200g less from those of older mothers) and smaller head circumferences.  For every kilogram increase in maternal weight, birth weight increased significantly by 8.08 grams.  For every 1 gm/dl fall in Hb levels, birth weight fell by 7.9g.  There were similar trends for crown heel length and placental weight, but not statistically significant. |
| 46 | 51 | Martin TC, Doyle B: **Decreasing prevalence of adolescent births in Antigua and Barbuda, 1969 to 1998 [abstract].** *West Indian Med J* 2000, **49**(Suppl. 2):53. | 90% of deliveries at Holberton Hospital, Antigua | Analysis of hospital records to determine trends of teenage pregnancy at Holberton Hospital, Antigua from 1969 -1998.  Births to mothers <18 and 18—19 yrs old were analysed separately  Yearly birth totals for each 5-yr period were compared using Chi Sauare analysis | Average of 1081 births per year  Females 12-17 yrs:  1969-1983 the no. of deliveries were 161-166  1983-1988 the no. dropped to 116 (p<0.01)  1989-1993 dropped to 90 (p=NS)  1994 – 1998 to 76 (p<0.02)  The percent decrease was 54% from 1969-1973 to 1994-1998.  Females 18-19 yrs:  From 1969-1983 the no. was 163-169 per year.  1983-1988 the no fell to 140 per yr (p<0.05)  1989-1993 to 106 per year (p<0.01)  1993-1998 111 per yr.  Percent decrease was 33% from 1969-1973 and 1994-1998  For all teenage deliveries, the percent decrease was 43% from 1969-1973 and 1994-1998  Per capita GDP rose by 43% from 1982 - 1998 | Not stated. |
| 47 | 52 | Family Planning Association of Trinidad and Tobago: **Results from the demographics and health survey 1987**. *Stud Fam Plann* 1989, **20**(4):235-239. | 3,806 women. | Interviews | Total fertility fell from 5.2 (1960) to 3.1 (1987)  In 1987 – little difference between urban and rural.  Lowest fertility in women with most education (2.3) vs. 3.6 with those with no education  Age specific fertility decreased over time, e.g. in 20-24 age group it fell from 306 (1967) to 181 (1987)  Women in the youngest group wanted the least amount of children 2.5 vs. 3.5 for 35-39 yrs olds.  Those with the least no. of children wanted the least no. / 2.5 for those with 0 children and 4.1 with those with 6 or more.  Total female sterilization stood at 8.3%/women with the most children had the highest rate – 25.4%  42.1% pregnant women with children either did not want any more children or wanted a child later. | 50% used contraceptive  Contraceptive use increased with education  - 40.9% of women with no primary school education used contraceptives vs. 67.6% of those with.  14% used oral contraceptive, 11.8% used condoms  Contraceptive prevalence ranged from 42.4% for 15 – 19 yrs olds to 57% for 30-34 yrs olds.  It increased with the number of children until the 3^rd^ child.  Government health centres and pharmacies distributed most of the contraceptives.  34.3% not using a contraceptive and in a union intended to use a contraceptive in the future  Reasons for not using contraceptive include no partner (25.7%), health concerns (21.2%)  Non educated women married earlier (17.1 yrs) than educated women (22.5%)  The mean length of breast feeding 10.1 month.  Infant mortality fell from 62 to 26% from 1960 -1987.  Trained nurses of midwives helped with most births |
| 48 | 53 | Roopnarinesingh S, Ali A, Bassaw B: **Is adolescent pregnancy hazardous?** *West Indian Med J* 1993 Mar, **42**(1):22-23. | 3,320 teenage mothers <20years at Mount Hope Women’s Hospital, Trinidad. | Retrospective case notes review between Jan 1988 and Dec 1991. | The perinatal loss was 2.2% and there was 1 maternal death.  Eclampsia rate of 540/100 000 deliveries.  Maternal mortality of 30.3/100 000 deliveries.  5.4% of infants required intensive neonatal care for prematurity and respiratory distress.  Caesarian section was required in 3.8%.  16.3% had low birth weight babies (< 2500 grams) | Not stated. |
| 49 | 54 | Kondamudi VK, Bhattacharyya A, Noah PK, Noel D: **Adolescent pregnancy in Grenada.** *Ann Trop Paediatr* 1993, **13**(4):379-383. | Adolescent deliveries 1987-1988  Maternal age 12-19 years | Review of using the delivery room register and hospital records  Compared with data of women between 20-30 yrs old. | Of 3,203 deliveries during the study period 613 (20%) involved adolescents – prevalence rate of one in five pregnancies.  Test revealed that the pregnancies in younger adolescents (<16 yrs old, n=55) carried an increased risk of preterm labour, operative delivery, prematurity, small for gestational age, infants, asphyxia and perinatal mortality.  Older adolescents (16-19 yrs, n =555) had a higher risk of pregnancy induced hypertension but compared well with the optimal reproductive age group  Adolescent pregnancy is very prevalent in Grenada and the reproductive outcome for adolescents < 12 yrs of age is relatively poor. | Not stated. |
| 50 | 55 | Pitter YJ: **Factors affecting outcome of pregnancy in adolescents at Victoria Jubilee Hospital in Kingston from period December 1st 1995 to February 29th 1996**. *Thesis.* Kingston: The University of the West Indies; 1996. | 570 Adolescents pregnancies at Victoria Jubilee Hospital Kingston/St. Andrew. | Questionnaire, checklist and interviews. | 77.5% first pregnancy  22.5% second pregnancy  94.6% SVD  4.5% C/S  0.7% Forceps  0.2% Misc.  0.2% Breech | Complications  Antepartum hemorrhage 0.9%  Postpartum hemorrhage 3.5%  Elevated Blood Pressure 16.3  Pre-eclampsia 3.2%  Eclampsia 1.1%  Premature rupture of membranes 2.5%  Prolonged lab 0.9% |
| 51 | 56 | Roopnarinesingh S: **Maternal mortality at Mount Hope Women's Hospital, Trinidad**. *West Indian Med J* 1991, **40**(3):139-141. | 57 ,012 live births over a ten-year period 1981-1990 | Retrospective case notes review. | Women aged 15-19 had one death and a maternal death rate of 1.75/100 000 births | Not stated. |
| 52 | 57 | Ramsewak S, Narayansingh GV, Sieunarine B: **Is teenage pregnancy high risk? - the experience in Trinidad [abstract].** *West Indian Med J* 1993, **42**(Suppl. 1):15-16. | 147 mothers >16 yrs delivering bet Jan 1 – Mar 31 1992 (2 – 13 yrs old, 14% were 14 yrs old and 16% were 15 yrs old)  145 control mothers 20-25 yrs old at the same time | Case-controlled study | Antenatal care was high (97%) for both groups.  The incidence of anaemia, pregnancy-induced hypertension, preterm labour and preterm rupture of the membranes was similar in teenagers and controls.  Gestational age at delivery was the same in the 2 groups.  Obstetrical performance as measured by antenatal and intrapartum complications was similar in the 2 groups and there was no significant difference in foetal outcome.  Unusual outcome – link to advanced status of T&T  Suggest that it is not necessary for pregnant teenagers to be referred for routine hospital ante natal care, thus allowing high-risk parturients of any age to be accommodated instead. | Not stated. |
| 53 | 58 | Venema AA, Datta B, Musket FAJ, Boersma ER: **Outcome of teenage pregnancy in St. Vincent: the adequacy of milk from teenage mothers in relation to neonatal growth [abstract].** *West Indian Med J* 1993 Apr, **42**(Suppl.1):57. | 33 randomly selected women (14-25 yrs old)  3 groups  (Group 1:  N=10, age 14-16 yrs old)  (Group 2: n=12 age 17-18 yrs) (Group 3 n=11 age 19-25) | Longitudinal study of pregnancy, neonatal outcome and lactation performances of adolescent and adult primagravidae using detailed data on obstetrical and neonatal (neurological) outcome.  After birth information was obtained on early infant growth, maternal dietary information were collected 2 times during home visits together with neonatal anthropometric measurements | No significant differences between the 3 groups in obstetrical conditions, birth weight and early infant growth were found.  In the youngest group, the number of infants diagnosed as neurological “suspect” was higher than those of the older age group.  Regarding analysis of breast milk: apart from lactose content, there were no major differences in the composition of the macro-nutrients;  No major differences in the fatty acid content, although a small no. of individual fatty acids were different between the groups. | It is concluded that previously reported perinatal problems of healthy teenage primagravidae (14-16 yrs old) are not substantiated in this series |
| 54 | 59 | Drayton VLC, Montgomery SB, Modeste NN, Frye-Anderson BA, McNeil P: **The impact of the Women's Centre of Jamaica Foundation programme for adolescent mothers on repeat pregnancies**. *West Indian Med J* 2000 Dec, **49**(4):316-326. | A random sample of 260 females b/w ages 15-20 who had first birth in 1994 and interviewed in 1998. | Historical cohort study design. 87 participants had an intervention vs. 173 non-participants. Participants completed a pre-tested questionnaire. | Not stated. | The risk predictors of one or more repeat pregnancies were common law relationships with either the father of the first baby or another current partner, perceptions of one’s socioeconomic status as very poor or poor and being a member of household where the respondent or spouse was the main wage earner.  Variables that exerted a protective effect against the occurrence of one or more repeat pregnancies were: participation in the intervention, the desire to continue one’s education after the birth of first child in 1994, taking action to continue education, use of contraception after first birth, being a member of a household in which the mother was the major wage earner at the time of the first birth and the absence of a current sexual relationship with their 1^st^ ‘baby father’ |
| 55 | 60 | Norman LR, Uche C: **Prevalence and determinants of sexually transmitted diseases: an analysis of young Jamaican males**. *Sex Transm Dis* 2000 Mar, **29**(3):126-132. | Male adolescents and young adults in Jamaica | Survey. | Overall, 9% of the sample reported symptoms of STDs in the year before the interview | Not stated. |
| 56 | 61 | Voisin DR, Dillon-Remy M: **Psychocultural factors associated with HIV infection among Trinidad and Tobago adolescents**. *Journal of HIV/AIDS Prevention & Education for Adolescents & Children* 2001, **4**(2-3):65-82. | 15-24 yrs olds in Trinidad and Tobago. | In-depth interviews with 10 females. | Not stated. | This paper reiterates that it is the cultural norm for men in Trinidad and Tobago to have 2 or 3 partners, that HIV infection has become more widespread as a result of increasing sex tourism. A paucity of information about sexuality and HIV in the media and stigma associated with HIV/AIDS are highlighted. |
| 57 | 62 | Adams OP, McIntyre G, Prussia P: **Risk behaviour, healthcare access and prevalence of infection with *Chlamydia trachomatis* and *Neisseria gonorrhoeae* in a population based sample of adults in Barbados [abstract]**. *West Indian Med J* 2004, **53**(Suppl. 2):18. | 321young people 18-35 yrs olds in St. Michael, Barbados. | Cross-sectional survey to determine the risk factors, health care access, symptoms and prevalence of urogenital infection with *Chlamydia trachomatis and Neisseria gonorrhoeae.* Participants randomly selected. | Overall prevalence of infection was s14.3% ( 95%, CI 10.5, 18.2  Prevalence of chlamydial infection was 12.8% and of gonococcal infection was 2.2%.  Prevalence similar in men and women. | Younger persons – more likely to be infected (18-21 yrs olds -26.4%)  21-35 yrs olds – 10.9%  Tertiary educated persons less likely to be infected.  29.4% of persons heard of chlamyida  91.3% had heard of gonorrhoea  Infection mostly asymptomatic in men |
| 58 | 63 | Walker E, Mayes B, Ramsay H, Hewitt H, Bain B, Christie CD: **Socio-demographic and clinical characteristics of Jamaican adolescents with HIV/AIDS.** *West Indian Med J* 2004 Oct, **53**(5):332-338. | 25 HIV-positive Jamaican adolescents, 10-19 yrs old | Characterization of clinical symptomatology and socio-demographic factors | Mean age of HIV diagnosis – 15.6 (+3.09) | Consensual sexual intercourse – most prominent mode of transmission – 56%  Vertical transmission – 16%  Unknown – 16%  Forced sexual intercourse- 8%  Blood transfusion – 4%  Provide reproductive health services, low cost/free condoms,  Voluntary counselling and testing.  Diagnosis and treatment of STD and infections for adolescents in order to prevent HIV infections. |
| 59 | 64 | Robinson T, Thompson T, Bain B: **Sexual risk taking behaviour and HIV knowledge of Kingston's street boys.** *Journal of HIV/AIDS Prevention & Education for Adolescents & Children* 2001, **4**(2/3):127-145. | Boys aged 13-17 | Focus group interviews using pre-developed questionnaire to guide the discussion. | Not stated. | Peer pressure and pressure by girls to have sex were identified by the respondents as to why they had sex. Most males used alcohol, to get a ‘buzz’, ‘to feel nice’ or ‘not to feel odd (different)’. Misconceptions included that many boys believed that they could identify a person with HIV, HIV feeds on blood vessels and that HIV was transmitted by mosquitoes. |
| 60 | 65 | Ministry of Health, Jamaica. **Report of the National knowledge, attitudes, behaviour and practices (KAPB) survey year 2004**. Kingston: Jamaica Hope Enterprises; 2004. | 1,498 persons, with 989 youth 15-24 years old. | Survey using a stratified multi-staged sample with quota controls for gender. | 56% of both genders perceive themselves not to be at risk of HIV infection.  16% of males and 32% of females had had an HIV test  Median age of first sex was 20 for males and 22 for females.  56% of males and 16% of females had had multiple partners in the past 12 months.  74% of youth reported using safe sex practices in high risk situations.  Among those reporting sexual activity in the past 12 months, 31% of both genders had had unprotected sex with a non-marital, non-cohabitating partner. | Not stated. |
| 61 | 66 | Ministry of Health,, Barbados. **Report of the National knowledge, attitudes, behaviour and practices survey on HIV/AIDS**. St. Michael, Barbados: Ministry of Education, Youth Affaires and Sport.; 2001 | 1,132 youth 15-29 years old. | Survey using a multi-stage random sample with interlocking quotas for age and gender. | 61% considered themselves unlikely to contract HIV  83% admitted to ever having sexual intercourse  Median age of first sexual intercourse was 16 years.  35% of males and 18% of females had had multiple partners in the past 12 months  31% used condoms always and 46% used condoms sometimes. | Not stated. |
| 62 | 67 | Ministry of Health: Trinidad and Tobago. **Youth response survey: a national survey of knowledge, perceptions and practices among 1500 youth in Trinidad and Tobago, subsequent to information, education and communication/ counselling activities on AIDS.** Port-of-Spain: Ministry of Health; 1995. | 1,500 youth in Trinidad. | Survey using youth interviewers involving clusters selected according to the case rates of AIDS in each county in Trinidad. | 57% felt they were not at risk for HIV infection  56% responded that they had had sexual intercourse.  Median age of first sexual intercourse was 16 years.  33% responded that they were using condoms to prevent HIV.  38% had had sex with one or more partners other than their regular partner in the last 12 months | Not stated. |
| 63 | 68 | Figueroa JP, Ward E, Luthi TE, Vermund SH, Brathwaite AR, Burk RD: **Prevalence of human papillomavirus among STD clinic attenders in Jamaica: association of younger age and increased sexual activity.** *Sex Transm Dis* 1995 Mar-Apr, **22**(2):114-118. | 202 women | Cross-sectional survey of women attending an STD clinic in 1990 | Prevalence of HPV by age group was  39% in women 15-19 years old,  33% of women 20-24 years old,  31% in women 25-29 years old,  17% in those 30 years or older. | Increasing age was significantly associated with a lower prevalence of human papillomavirus infection (test for trend, P = 0.025).  The effect of age was independent of years of sexual activity. Women reporting more than one sexual partner per month on average were found to have a significantly higher HPV prevalence (odds ratio 2.87, 95% confidence Interval 1.29-6.38), as were women who reported more frequent sex (test for trend, P = 0.006) |
| 64 | 69 | Payne MA: **Adolescent fears: some Caribbean findings**. *J Youth Adolesc* 1988 Jun, **17**(3):255-266. | 657 adolescents (12-15 yrs) in Barbados | Questionnaire – Is there anything you are really scared or afraid of? | 3 main categories  1. fear of injury or death of self or loved ones,  2 sexual concerns  3 fear associated with school failure | Need for improved sex and family life education |
| 65 | 70 | Hickling FW: **The psychopathology of children and adolescents in Jamaica [abstract]**. *West Indian Med J* 1993 Apr, **42**(Suppl. 1):48. | 194 children < 19 yrs old or less in Jamaica. | Survey of patients seen in a psychiatric clinic. | - 4% retarded (8 patients < 10 yrs old)   Of the other 186 patients   - 28% - adolescent crisis (mainly females) - 22% - schizophrenia (mainly males) - 18% - affective disorder | - Parental conflict - Parental hostility - Psychosexual problems   Avoid   - Parental separation - Divorce - Absence of one parent |
| 66 | 71 | Mahy GE: **Parasuicide in Barbados**. *West Indian Med J* 1980, **29**(28-33). | 168 patients at Queen Elizabeth Hospital in Barbados. | Review of all cases of attempted suicide, 1974-1977. | Adolescents represented 34/168 of attempted suicide.  7.5:1 (Female to Male). | Not stated. |
| 67 | 72 | Marceau-Crooks H, Jinkson J, Roberts G: **Parasuicides in South Trinidad - 1974 and 1984 [abstract]**. *Caribb Med J* 1992 Sep, **53**(Suppl. 1):8. | 628 cases | Questionnaire administered by psychiatrist or mental health officer | 75% were adolescents  and young adults. | Not stated. |
| 68 | 73 | Neehall JE, Beharry N: **Demographic and clinical features of adolescent parasuicides**. *West Indian Med J* 1994, **43**(4):123-126. | 102 adolescents admitted to the Port-of Spain General Hospital over a 10-month period | Interviews, case-notes review and mental status examination | The estimated rate of attempted suicide among adolescents was 94 per 100,000 of the hospital catchment population.  90% of sample was female.  25% was depressed and 22% had adjustment disorders. | Indo-Trinidadian females were more frequent attempters than their Afro-Trinidadian or mixed counterparts.  Intra-familial and ‘lovers’ conflict was the most common reason given for attempted suicide. |
| 69 | 74 | Dookhan DA: **Self poisoning in the adolescent and adult population at the Georgetown Hospital, Guyana [abstract]**. *West Indian Med J* 1997 Apr, **46**(Suppl. 2):45. | 168 admissions for self-poisoning | Review of admissions registers of the Emergency Unit at the Public Hospital, Georgetown | 114 East Indians  53 Africans  1 Chinese  Parasuicidal rates:  55/100 000 –  Adults  57/100 000 – Adolescent  East Indian: African attempters ratio  2:1  Population ration 1.5:1  79 males:89 females  10-19 age group: 52 cases  20-29 age group: 61 cases  30-39 age group: 28 cases  40-59 age group: 13 cases  The youngest: 12 yrs old/ the oldest – 90 yrs old.  141/168 or 84% were in the age 10-39 yr group.  An average of 14 cases a month  Substances  Organophosphates – 58 cases  Household substances – 40 cases  Medicinal Tablets – 26 cases  Kerosene oil – 25 cases  Gramoxone – 10 cases | Preventative measures, including education, should be targeted to farmers and youths and young adults in the 10- 29 age group. |
| 70 | 75 | Sinanansingh P: **A study of parasuicide among adolescent and young adult in southern region of Trinidad**. *Thesis.* Kingston: s.n.; 1997. | 75 parasuicides visited at home in South Western region | Pre-tested Questionnaire | 75% were adolescents.  East Indian predominance  F:M =2:1 | Risk and Precipitating factors.  Family instability 40%.  Emotional problems 29 6%  Financial difficulties %24  Peer pressure 22%  Conflict with parents and family members 38.4%  Unemployment12%  Quarrel1 2% |
| 71 | 76 | Sharma KLD: **Analysis of suicide attempts in the commonwealth of Dominica [abstract]**. *West Indian Med J* 1998 Apr, **47**(Suppl 2):56. | An analysis of all suicide attempts between 1994 and 1997 in Dominica. | A consultation-liaison register of largest island hospital. | Total population rate of 37/ 100 000  Adolescents below 18 years made up 53% of cases.  Females made up 73% of all cases and 81% of all adolescents. | Not stated. |
| 72 | 77 | Pottinger AM, Milbourn PE, Leiba J: **Suicidal behaviour and risk factors in children and adolescents in Jamaica**. *West Indian Med J* 2003 Jun, **52**(2):127-130. | 57 children and adolescents attending 4 clinics in metropolitan Kingston. | Case record review of patients seen b/w 1998 and 1999 who exhibited suicidal behaviour | 41,/57 were 12-18y | Attempted suicide and self harm associated with having a poor relationship with primary care taker (72%) and experiencing abuse (55%) |
| 73 | 78 | Hutchinson G, Daisley H, Simmons V, Gordon AN: **Suicide by poisoning**. *West Indian Med J* 1991 Jun, **40**(2):69-73. | 270 deceased b/w Jan 1986- June 1990. | Autopsy and toxicology analysis | 10% in age group 11- 18 years  25% in age group 9-26 years | Not stated. |
| 74 | 79 | Hutchinson G, Daisley H, Simeon D, Simmonds V, Shetty M, Lynn D: **High rates of paraquat-induced suicide in southern Trinidad**. *Suicide Life Threat Behav* 1999, **29**(2):186-191. | 48 cases for the year 1996 | Review of records where death was due to suicide | 39 (81.3%) due to paraquat poisoning  i.e. 8 per100,000  Among males – 47.8% were 25-34 yrs old (p<0.001)  Among females  50.0% were 15-24 yrs old (p<0.05)  89% East Indian | Family –of-origin disputes  Marital problems  Being a Young East Indian |
| 75 | 80 | Daisley H, Simmons V: **Forensic analysis of acute fatal poisonings in the southern districts of Trinidad**. *Vet Hum Toxicol* 1999 Feb, **41**(1):23-25. | 105 deaths from acute poisoning | Study of autopsies to analyze deaths from poisoning | Paraquat – 80 cases  Organophosphate/carbamate insecticides – 10 cases  Anti-psychotic drugs – 6 cases. Other – battery acid, ethrel, kerosene, flavine, ethanol etc.  99 were suicide  Of these, 44.44% were in the 10-29 yr age group | Not stated. |
| 76 | 81 | Ali A, Maharajh HD: **Social predictors of suicidal behaviour in adolescents in Trinidad and Tobago**. *Soc Psychiatry Psychiatr Epidemiol* 2005 Mar, **40**(3):186-191. | 1 845 respondents aged 14-20 y in Trinidad and Tobago. | A random sample of schools stratified by counties and drawn to represent the county size. 21 schools were drawn from Trinidad and 3 from Tobago. Self-administered Suicidal Ideation Questionnaire. | Attempted suicide rates for different family structures were:  Intact families- 6.1%  Living with relatives- 8.3%  One-parent family- 9.6%  Reconstituted family- 14.6%  Single step-parent family 16.7% | Females had greater rates of suicidal ideation and suicidal attempts (F 9.8%: M 4.5%).  Individual who attended a religious institution more than 7 time sin the past 6 months had significantly lower suicidal ideation than those without attendance at a religious institution at all over the same time period.  Individuals who had never prayed with their families had higher rates of suicide attempts than individuals who prayed with their families.  Individuals from reconstituted families had the greatest suicidal ideation.  Alcohol use and family alcohol abuse was associated with both suicidal ideation and suicidal attempts. |
| 77 | 82 | Maharajh HD, Ali A: **Depression in Tobagonian adolescents**. *Int J Adolesc Med Health* 2004, **16**(4):337-342. | 203 adolescents aged 14 -18 years  85% African descent; 99% Christians | Self-administered Suicidal Ideation Questionnaire. | 10.1% of respondents were depressed. | Females more depressed |
| 78 | 83 | Roopnarinesingh N, Alli F, Cumberbatch K, Laloo P, Mohammed S, Ramesar A, Rampersad N, Maharaj RG, Ramtahal I: **The prevalence of depression among adolescents attending secondary schools in Trinidad [abstract]**. *West Indian Med J* 2004, **53**(Suppl. 2):76. | 1,290 students aged 13-19. 43% were aged 13-15 years, 53.6 % were Indo-Trinidadian, 82.5% were attending co-educational schools and 70.6% lived with both parents. | A cross-sectional study of a stratified random sample of public secondary schools utilising a modified pre-tested self-administered Beck Depression Inventory (BDI) to detect depression among | The prevalence of depression was 25.3% +/- 2.37%. | Chi-square analysis revealed statistically significant associations between depression and the categories of age, gender, living arrangements and school type. Similar findings were observed for respondents who admitted to cigarette and alcohol use or to being afraid of, or being injured by their parent, (p < 0.05). Logistic regression indicated that females were 1.7 times as likely to be depressed when compared with males; respondents not living with both parents were 1.5 times *as* likely to be depressed *as* those who were. Respondents reporting that they were afraid of parents or being injured by parents were 3 times *as* likely to be depressed *as* respondents who had not had those experiences. |
| 79 | 84 | Lowe G, Lipps G, Abel W, Brown A, Hickling FW: **Depression among fourth form students in three high schools in Kingston [abstract]**. *West Indian Med J* 2005, **54**(Suppl. 2)):62. | 457 form 4 students (age range 14-18 years) | Screening of 3 high schools in Kingston, Jamaica using Beck’s Depression Inventory | Overall rate of 60.4%, with severe depression being 9%. 32.2 % admitted to suicidal ideation, but only 5.5% reported that they would attempt if given the opportunity. |  |
| 80 | 85 | Patrickson L: **Factors contributing to the juvenile delinquency among girls in Jamaica**. *PhD. Thesis.* Kingston: The University of the West Indies; 1996. | 33 juvenile females 13 – 18 yrs  companion groups (121-n) from Kingston secondary school. | Case controlled study. Questionnaire | Not stated. | Risk Factor contributing to juvenile delinquency   - - Absence of father (OR 3.5 (95%CI 1.14-11.03))   - Violence in the home (OR 2.76 (95% CI 0.09-8.64))   - Drug use and abuse   - Associated with gangs   - Separated from mother (OR 4.69 (95%CI 1.5-20.59)). |
| 81 | 86 | Cosentino R: **Social, economic and cultural factors contributing to adolescents dropping out of school in Grenada, West Indies.** New York: Columbia University. School of Public Health; 1994. | Not stated | Review of available qualitative data,  In-depth interviews with persons providing services to teens, school leavers and drop-outs | The most common reasons were breakdown in family structure; economic factors, such as, barriers within the educational system and customs and culture. | Not stated. |
| 82 | 87 | Perks SM, Jameson M: **The effects of witnessing domestic violence on behavioural problems and depressive symptomatology. A community sample of pupils from St. Lucia**. *West Indian Med J* 1999, **48**(4):208-211. | 60 students who came from homes where parents were either in violent or non-violent relationships. | Teacher rating of student using the Revised Behavior Problem Checklist and students self rated their depression using the Reynolds Child or Reynolds Adolescent Depression scale, depending on their age. | Pupils whose parents were undergoing violent marital discord were more likely to be depressed or to have behavioural problems. | Not stated. |
| 83 | 88 | Kirsch TD, Beaudreau RW, Holder YA, Smith GS: **Pediatric injuries presenting to an emergency department in a developing country.** *Pediatr Emerg Care* 1996 Dec, **12**(6):411-415. |  | 14-day surveys of all injuries were conducted in the Emergency Departments of the two national trauma referral hospitals in Trinidad and Tobago. | Pediatric patients (<20 yrs old) accounted for 41.5% of injury visits. Of these, 62.6% were male and 17.4% were <4 yrs old, 26.2% were 15-19 yrs old.  3 patients (0.4%) died, 68.6% were discharged, and 31.0 % were admitted.  There were 13. 9% intentional injuries.  Of the intentional injuries, the assailant was significantly more likely to be known than not (p<0.01). The most common causes of all injuries were falls (44.4%); blunt objects, 12.3%; sharp objects, 11.8%; motor vehicle (including pedestrians), 7.4%; poison 3.6%; and burns 1.7%.  Injuries occurring in the home accounted for 46.2%; in school, 25.5%; sports/recreation, 11.1% and at work, 4.5%.  The most common injuries were: lacerations, 30.8%; contusions/abrasions 26.7%; fractures, 18.8% and sprains/dislocations 9.4%. | Pediatric injuries are a significant cause of morbidity and mortality in this country, accounting for almost one third of injured patients. |
| 84 | 89 | Noah P, Rao MR, Weis U: **Adolescent admissions in Barbadian hospitals [abstract].** *West Indian Med J* 1993 Apr, **42**(Suppl. 1):26. | 725 admissions – 297 males and 428 females  535 (74%) spent 1-5 days and only 55 (7.6%) spent >14 days in hospital | Retrospective review of adolescent admissions (12-18 yrs old) from Oct 1991 – Sept 1992 | 3 most frequent causes for hospitalization were trauma (165), abortions (153) and drug abuse/overdose (53). | Not stated. |
| 85 | 90 | Whittle S: **Knowledge about causes, effects, attitudes towards and experiences with violent acts among students in secondary schools, Jamaica**. *MPH Thesis.* Kingston: The University of the West Indies; 1994. | 748 Grade 9 & 10 students from two high schools in Jamaica. | Random selection of students with a pre-tested Questionnaire. Group discussion.  Interview with key informants | 20% of sample had experienced violent acts. | 1. No relationship between age, sex, student’s grade, family structure or employment status & violence. 2. Student’s whose fathers had higher education levels (≥ 2^0)^ tended to be more violent. (p < 0.05) 3. Students with gas stores or motor bikes more violent (p < 0.05) |
| 86 | 91 | Soyibo K: **Domestic and school violence among high school students in Jamaica**. *West Indian Med J* 2000, **49**(3):232. | 3124 secondary school students aged 16-17+ | Students from 34 randomly selected high schools surveyed with pre-tested questionnaire | 78.5% had witness violence in the community.  44% at home.  60.8% at school.  29% had caused harm to others.  24% experienced injury in domestic violence.  38.9% had a family member as a victim of domestic violence  Between 5-27% had used a weapon during an act of violence | Not stated. |
| 87 | 92 | Meeks Gardner J: **Perceptions and experience of violence among secondary school students in urban Jamaica**. *Pan Am J Public Health* 2003, **14**(2):97-103. | 1710 students 9-17 y | Survey in 11 schools randomly selected to represent different types of secondary schools; carried out through researcher administered pre-tested (test-re-test 0.78) questionnaire. | 33% had been a victim of violence.  60% had family member who had been a victim of violence. | Boys, older students and those with poorer socioeconomic status reported higher neighbourhood violence.  Boys and students from higher socioeconomic status reported higher levels of school violence.  Students from all age schools/ junior high schools reported higher neighbourhood and school violence as compared with students from technical/comprehensive high schools.  Attendees at all age/junior high school had high concerns about violence and greater experiences and perceptions of violence than those attending other school types. |
| 88 | 93 | Samms-Vaughn ME, Jackson MA, Ashley DE: **Urban Jamaican children's exposure to community violence**. *West Indian Med J* 2004, **54**(1):14-21. | 1720 11-12 year-olds, a geographic sub-group (Kingston and St. Andrew parishes). | Children were identified during the Jamaican perinatal mortality and morbidity study (a national birth cohort of 10 000 children identified in 1986-7 by their date of birth from the Ministry of Education and school records). Parents and children were interviewed at school by trained interviewers. | The 6 leading lifetime exposures to specific violent acts included fights (92.5%), stoning (65.7%), arrests by policemen (60.4%), serious threats (54.6%),, viewing of dead bodies other than at funerals (45.6%) and stab-wounding (43.9%).  Children most often reported being victims of fights (34.8%), stone-throwing (23.4%), threats (22.3%), robbery 919.4%), and stab-wounding (12.6%).  73.4% had been in a fight over their lifetime. 8.1% reported carrying a weapon during their lifetime. | Children from the highest socio-economic groups had a much lower lifetime prevalence of witnessing all violent activity, but were just as likely as lower economic groups to experience threats or robbery.  Children from lower socio-economic groups were more likely to experience beatings, stone throwing, and stabbings.  Multivariate analysis revealed that boys and primary school children were more likely to witness violence and be a victim of violence and children residing in lower socio-economic households were more likely to witness violence but had no greater likelihood of being a victim. |
| 89 | 94 | Mahy GE: **Anorexia nervosa in Barbados [abstract]**. *West Indian Med J* 1991, **40**(Suppl. 2):118. | Not stated | Review of records to find out the prevalence of anorexia nervosa in the island of Barbados and to develop theories to explain the low prevalence. | When it occurs, the family characteristics are similar to those of the more developed western societies   Anorexia nervosa seems even more uncommon in the lower socio-economic group | Not stated. |
| 90 | 95 | Bhugra D, Mastrogianni A, Maharajh H, Harvey S: **Prevalence of bulimic behaviors and eating disorders in schoolgirls in Trinidad and Barbados**. *Transcultural Psychiatry* 2004, **40**(3):410-428 | 362 schoolgirls | Survey and interview of randomly chosen classes from 4 schools 2 each in Trinidad and Barbados. | 0.8% positive on bulimic scale but not confirmed as bulimic. | Not stated. |
| 91 | 96 | McGuire MT, Story M, Neumark-Sztainer D, Halcón, L, Campbell-Forrester S, Blum RW: **Prevalence and correlates of weight-control behaviors among Caribbean adolescent students**. *J Adolesc Health* 2002 Aug, **31**(2):208-211. | Caribbean Youth Health Survey (n= 15 695). African 78.5%, East Indian 6.5%, Amerindian 5.5% descent.  Mean age = 13.7 yrs | Data from an anonymous survey of 9 countries in Anglophone Caribbean using a pre-tested 87-item pencil and paper instrument derived from the Minnesota Adolescent Health Survey and the Youth Risk Behavior Survey. | Weight-control behaviours were prevalent and similar across genders | Extreme weight control behaviours were related to several psychosocial factors and compromising health behaviours. Future interventions should target adolescents who are using weight-controlling behaviours to prevent future eating disturbances and pscyhosocial and health morbidities. |
| 92 | 97 | Alert C, Broome H, Holland A, Mellanson-King R, Fraser HS: **Physical activity in Barbadian secondary school attenders - result from the Adolescent Health and Fitness study (AHFIT) [abstract]**. *West Indian Med J* 2000, **49**(Suppl. 2):26. | 462 students | A randomly selected sample of students from 4 schools. A KAP (knowledge, attitudes and practices) questionnaire applied and sample of blood taken. | 15% (8% males and 20% females) of students aged 10-18 did not participate in any form of physical activity on a regular basis | Not stated. |
| 93 | 98 | Gaskin P, Broome H, Alert C, Griffith B, Fraser HS: **Adolescent obesity, disproportionately affects girls in Barbados [abstract]**. *West Indian Med J* 2005, **54**(Suppl 2):44. | 164 boys and 236 girls, 11-16 year-old school children gathered to study physical education | Cross-sectional study conducted in Barbados | Results considered high :  Overweight (15% boys and 17% girls)  Obesity (7% boys and 12% girls) - | Maternal obesity predicted weight status: an obese mother increased odds of being overweight by 5.3 (95% CI:2.7, 10.5)  50% girls – no structured Physical activity  Overweight subjects tended to misclassify themselves as normal weight. |
| 94 | 99 | Simeon DT, Rattan RD, Panchoo K, Kungeesingh KV, Ali AC, Abdool PS: **Body image of adolescents in a multi-ethnic Caribbean population**. *Eur J Clin Nutr* 2003 Jan, **57**(1):157-162. | 1,139 adolescents aged 14-17y attending secondary schools in Trinidad | A stratified random sample of adolescents who completed a questionnaire; a cross-sectional survey | 40% associated male overweight and obese silhouettes with happiness.  73% had normal BMI  13% overweight | Not stated. |
| 95 | 100 | Lehman SC, Mahabir D, Modlesky CM, Lewis RD: **Prevalence of overweight in Trinidadian adolescents [abstract]**. *West Indian Med J* 2003 Jun, **52**(Suppl. 3):22. | 296 adolescents (12-18 years old) (126 males  170 females) attending secondary schools in Trinidad. | Survey of a random sample adolescents in Trinidad  Demographics, height, weight, three-site skin fold thickness, dietary intake using 3-day records, and physical activity recorded | 13.2% were at risk-for-overweight  4.4% were overweight  Females  15.9% risk-for overweight  7.1% overweight  Males  9.5% f risk-for-overweight  0.8%overweight  African females  18.8% risk-for-overweight  15.6% overweight  Africans had higher values for height, weight and BMI than Indians | Not stated. |
